# Effect of early measles vaccine on pneumococcal colonization: A randomized trial from Guinea-Bissau

**DOI:** 10.1371/journal.pone.0177547

**Published:** 2017-05-17

**Authors:** Nadja Skadkær Hansen, Stine Byberg, Lars Hervig Jacobsen, Morten Bjerregaard-Andersen, Aksel Karl Georg Jensen, Cesario Martins, Peter Aaby, Jørgen Skov Jensen, Christine Stabell Benn, Hilton Whittle

**Affiliations:** 1 Research Center for Vitamins and Vaccines (CVIVA), Bandim Health Project, Statens Serum Institut, Copenhagen, Denmark; 2 Bandim Health Project, INDEPTH Network, Bissau, Guinea-Bissau; 3 Department of Endocrinology, Odense University Hospital, Odense, Denmark; 4 Section of Biostatistics, University of Copenhagen, Copenhagen, Denmark; 5 Department of Microbiology and Infection Control, Statens Serum Institut, Copenhagen, Denmark; 6 Odense Patient data Explorative Network, Odense University Hospital/Institute of Clinical Research, University of Southern Denmark, Odense, Denmark; 7 The London School of Hygiene and Tropical Medicine, London, United Kingdom; Public Health England, UNITED KINGDOM

## Abstract

**Background:**

Measles vaccine (MV) may have non-specific beneficial effects for child health and particularly seems to prevent respiratory infections. *Streptococcus pneumoniae* is the leading cause of bacterial pneumonia among children worldwide, and nasopharyngeal colonization precedes infection.

**Objective:**

We investigated whether providing early MV at 18 weeks of age reduced pneumococcal colonization and/or density up to 9 months of age.

**Method:**

The study was conducted in 2013–2014 in Guinea-Bissau. Pneumococcal vaccine was not part of the vaccination program. Infants aged 18 weeks were block-randomized 2:1 to early or no early MV; at age 9 months, all children were offered MV as per current policy. Nasopharyngeal swabs were taken at baseline, age 6.5 months, and age 9 months. Pneumococcal density was determined by q-PCR. Prevalence ratios of pneumococcal colonization and recent antibiotic treatment (yes/no) by age 6.5 months (PR_6.5_) and age 9 months (PR_9_) were estimated using Poisson regression with robust variance estimates while the pneumococcal geometric mean ratio (GMR_6.5_ and GMR_9_) was obtained using OLS regression.

**Results:**

Analyses included 512 children; 346 early MV-children and 166 controls. At enrolment, the pneumococcal colonization prevalence was 80% (411/512). Comparing early MV-children with controls, the PR_6.5_ was 1.02 (95%CI = 0.94–1.10), and the PR_9_ was 1.04 (0.96–1.12). The GMR_6.5_ was 1.02 (0.55–1.89), and the GMR_9_ was 0.69 (0.39–1.21).

Early MV-children tended to be less frequently treated with antibiotics prior to follow up (PR_6.5_ 0.60 (0.34–1.05) and PR_9_ 0.87 (0.50–1.53)). Antibiotic treatment was associated with considerably lower colonization rates, PR_6.5_ 0.85 (0.71–1.01) and PR_9_ 0.66 (0.52–0.84), as well as lower pneumococcal density, GMR_6.5_ 0.32 (0.12–0.86) and GMR_9_ 0.52 (0.18–1.52).

**Conclusion:**

Early MV at age 18 weeks had no measurable effect on pneumococcal colonization prevalence or density. Higher consumption of antibiotics among controls may have blurred an effect of early MV.

**Trial registration:**

clinicaltrials.gov NCT01486355

## Introduction

In a recent WHO commissioned review[1], the live attenuated measles vaccine (MV) was associated with reduced overall child mortality[2, 3] and morbidity[4, 5] exceeding what is attributable to prevention of measles infection. This indicates non-specific effects (NSEs) of the vaccine. A Guinean randomized trial[4] and a register-based Danish study[6] both found that MV was associated with significantly fewer hospital admissions with respiratory infections when comparing measles-vaccinated children with controls whose most recent vaccination was DTP-HepB-Hib-OPV-3 and DTP-Hib-IPV-3, respectively.

A recent observational study[7] from The Gambia found that MV and yellow fever-vaccine (YF) co-administered at age 9 months reduced nasopharyngeal (NP) carriage of *Streptococcus Pneumoniae* (pneumococci) by 75% (OR 0.25 (0.07–0.90)) when comparing carriage status 4 weeks after vaccination with before.

Pneumonia is the single largest killer of children <5 years worldwide with more than 95% of cases occurring in low-income countries[8]. The most frequent bacterial cause is pneumococci.

Globally, pneumococci are estimated to cause 11% of all deaths among children <5 years with 90% of fatalities due to pneumonia[9]. Pneumococcal carriage is common among children in low-income countries from an early age[10, 11] and may be asymptomatic, but is also a prerequisite for pneumococcal disease development[12].

We hypothesized that MV decreases respiratory infections with *S*. *Pneumoniae* through an effect on the nasopharyngeal colonization. Thus, within an ongoing randomized controlled trial (RCT) of early MV among infants in Guinea-Bissau, we investigated whether MV reduced pneumococcal colonization and/or -density.

## Materials and methods

### Study setting

Since 1978, the Bandim Health Project (BHP) has maintained a health and demographic surveillance system (HDSS) currently covering more than 100,000 individuals in 6 suburbs in the capital of Bissau, Guinea-Bissau. Through monthly home visits newborns are identified and followed with trimonthly home visits until 3 years of age to register growth, morbidity, vaccinations and vital status. At the initial registration, each child is given a unique ID number to facilitate linkage between all BHP registers.

### Main early MV trial

The present pneumococcal study (MVPneumo) was nested within a RCT conducted within the BHP urban study area from 2011 to 2015, aiming at investigating the effect of early vs. no early MV at 18 weeks of age on overall mortality until age 5 years (MVUrban). All children were offered the recommended MV at age 9 months. Pneumococcal conjugate vaccine (PCV) had not been implemented in Guinea-Bissau when MVPneumo was conducted. There were no reported measles cases throughout the duration of the study.

Inclusion criteria in the MVUrban trial were receipt of the third dose of the pentavalent vaccine (DTP-HepB-Hib-3) at least 4 weeks before enrolment, and age below 7 months. Through home visits, eligible children were invited for inclusion at one of the three health centers in the HDSS study area. Here, children underwent a clinical examination by a physician and had anthropometric values measured. Severely sick children in need of hospitalization, malnourished children (defined as a mid-upper-arm circumference less than 115 mm) and children with severe malformations were excluded.

Provided informed oral and written consent from a parent/guardian was obtained, children were block-randomized 2:1 stratified by sex to early or no early MV at age 18 weeks. Parents picked a folded lot from a sex-specific envelope containing 24 lots, 2/3 of the lots assigned the child to the intervention group (early MV), 1/3 of the lots assigned the child to the control group (no early MV). Twins of the same sex were allocated to the same treatment to avoid confusion. Children randomized to early MV received a single dose of 0.5 ml MV (Edmonston-Zagreb strain, Serum Institute of India, Pune, India) as a subcutaneous injection in the shoulder region. No placebo was given, since mothers could get the false impression that their child was vaccinated against measles and therefore abstain from the recommended 9 months MV if they travelled or moved from the BHP study area.

### The pneumococcal sub-study

From August 2013 to January 2014, children enrolled in MVUrban were invited to participate in MVPneumo. Provided informed consent, three nasopharyngeal swabs (NPS) were scheduled to be taken: At enrolment, 2 months later (approximately age 6.5 months), and at 9 months of age. Children older than 6 months at enrolment were not followed up before age 9 months. The last child was followed up by June 2014.

Children and their caretakers were visited at home by field assistants and invited to the health center for NPS.

At the health center a questionnaire was completed comprising health status of the child, anthropometric values were measured, and the nasopharynx was swabbed. Information on recent medication of the child (within last three days) and the mother (within last week) was registered (antimalarials, antibiotics, pain killers, and others; if possible, the specific name(s) of medicine taken was registered).

At 9 months of age, the NPS was taken immediately before measles vaccination.

MVPneumo staff were blinded to the randomization status of children since randomization took place in a separate room after the first NPS was obtained. Moreover, randomization status was not registered on the health card of the child so the staff remained blinded at follow up visits.

All procedures of MVUrban and MVPneumo were carried out by employees of the BHP, including doctors, nurses and trained assistants.

#### Nasopharyngeal swabs

NPS procedures followed a protocol based on WHO recommendations[13]. A flexible swab (Minitip Flocked Swab, Copan, Brescia, Italy) was inserted to the level of the posterior nasopharynx[13, 14] and rotated. If resistance was met before this point, the swab was discarded and a new attempt made through the other nostril. If second attempt failed, no specimen was obtained.

The swab was then rotated in 0.5 ml 0.9% saline and a 0.25 ml aliquot of this specimen solution was transferred by a 1 ml single-use Pasteur-pipette to a cryotube containing 100 μl DNA stabilizing transport medium (AssayAssure, Sierra Molecular, Sonora, CA, USA) and shaken. Samples were temporarily contained in a cold box until frozen at minimum -20°C within a maximum of 7 hours. All equipment was disinfected twice with 70% ethanol between swabbing procedures and the staff wore disposable gloves within the process.

#### Detection of *S*. *pneumoniae* by quantitative PCR

Genomic DNA was extracted from 125 μl of the NPS specimen solution using the FastDNA^™^ spin kit for soil (MPBiomedicals, LLC, Santa Ana, CA, USA) with elution into 100 μl. The number of pneumococci detected from 5 μl DNA-extraction was determined using a quantitative real-time PCR (q-PCR) detecting the autolysin gene (*lyt*A) as described by the US CDC[15]. Furthermore, an internal amplification control was constructed as previously described[16]. PCR was performed in a 50 μl final volume with thermocycling conducted on an ABI 7500 real-time PCR instrument. A standard curve was constructed from 10-fold dilutions of purified *S*. *Pneumoniae* DNA in TE buffer containing calf-thymus DNA as stabilizer/carrier DNA[16]. Results were analysed using 7500 Software v2.3, Applied Biosystems. In case of absence of internal control the sample was re-analysed. All analyses were conducted at Statens Serum Institut in Denmark (PCR methods described in detail in the S1 Appendix). Laboratory technicians were blinded to the randomization status of children.

### Statistical analysis

All analyses were based on intention-to-treat. One mother agreed to participate in MVUrban but refused to let her child be vaccinated when randomized to early MV; however, estimates from per-protocol analyses were similar since reclassification of one observation did not alter the findings.

Categorical variables describing pneumococcal colonization status (No/Yes), were defined based on the presence of pneumococcal DNA detected by q-PCR. In addition, a categorical variable describing recent antibiotic treatment of the child (No/Yes) was defined. The analysis of the effect of antibiotics on pneumococcal colonization and density was not originally planned but was conducted after discovering that early MV children had a markedly lower consumption of antibiotics than controls.

The primary outcomes, pneumococcal prevalence ratios at 6.5 and 9 months of age (PR_6.5_ and PR_9_), were estimated using Poisson regression with robust variance estimation[17]. The secondary outcomes, geometric mean ratios (GMR_6.5_ and GMR_9_) of pneumococcal density according to MV status, were computed for colonized children. We applied linear ordinary least square (OLS) regression, using the logarithmic transformed density as variable.

#### Stratified analyses

In the protocol it was pre-specified that all analyses would be stratified by sex.

A previous study suggested that receiving oral polio vaccine (OPV) during campaigns prior to early MV is associated with a less beneficial effect of early MV[18], hence, we stratified the present data on whether children had participated in OPV campaigns prior to enrolment or not.

Moreover, in a Guinean study[4] investigating the effect of early MV on hospital admissions, the beneficial effect was strongest among children vaccinated in the dry season (Dec-May). Hence, we also stratified by season at enrolment.

Stratification by receipt of OPV and season were not pre-specified in the protocol, but based on the above mentioned findings.

#### Sensitivity analysis

Lower pneumococcal density values are related to increased measurement uncertainty, and only single assessments were done. Nonetheless, exploring cut off values of density ranging from 100 through 2,000 genome equivalents did not modify the estimate of the MV effect on colonization or density. Thus, a cut off was not implemented in the analyses.

All estimates are reported with 95% confidence intervals. Analyses were performed in Stata version 12.0 (StataCorp, College Station, TX, USA).

#### Sample size

We intended to include 420 early MV-children and 210 controls based on detecting a 10% reduction of pneumococcal colonization among MV-children, from 85% to 75%, with a power of 80% and significance level α = 0.05.

### Ethical considerations

The early MV trial and the pneumococcal sub-study were approved by the Ethical Committee in Guinea-Bissau (CNES-2013-031). The Developing-Country Committee of The National Committee on Health Research Ethics, Denmark, gave its consultative approval. All participants gave their informed consent. MVUrban is registered at clinicaltrials.gov NCT01486355

## Results

### Study population

**Fig 1:**
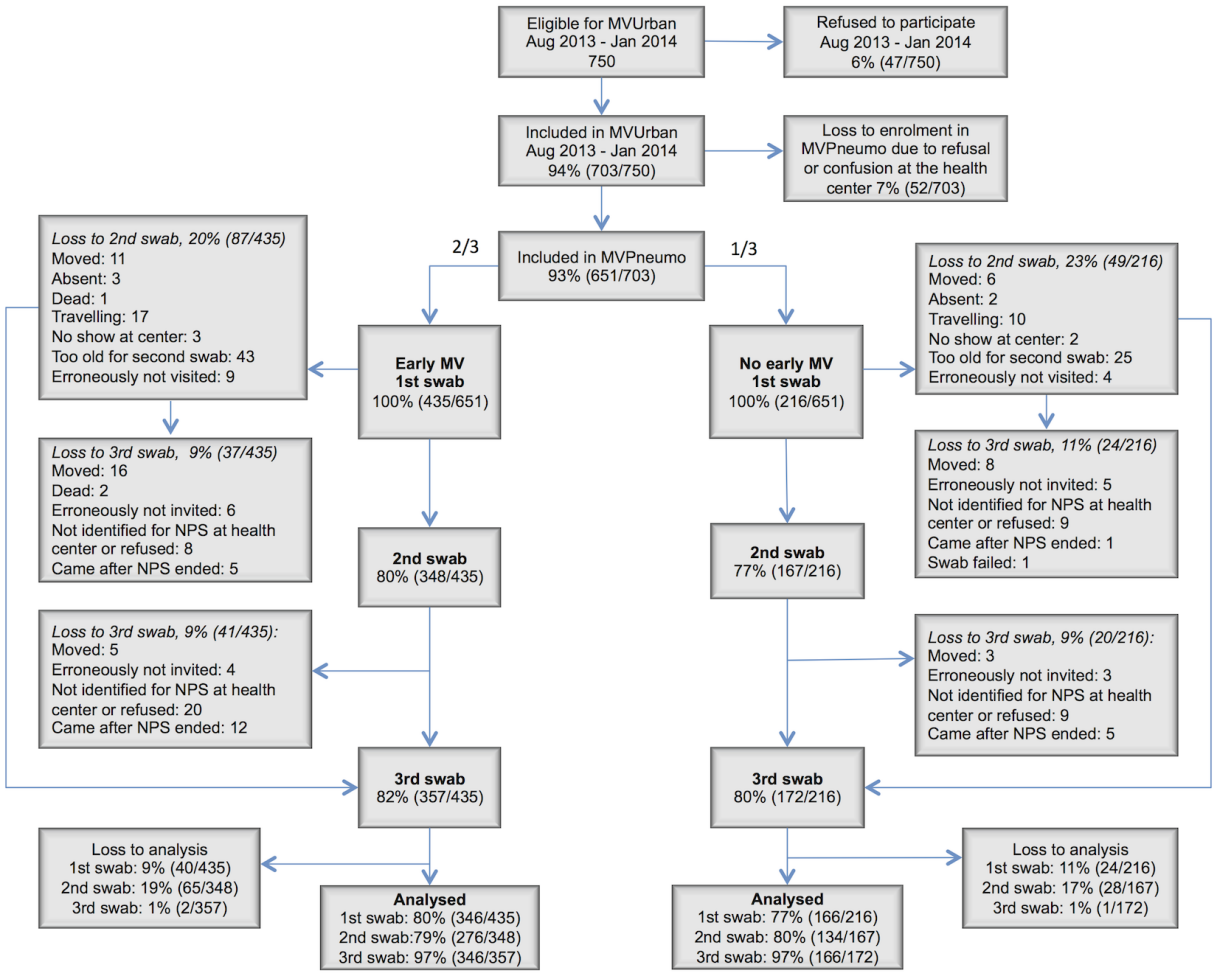
Flowchart of MVPneumo.

Within the pneumococcal study period, 703 children (94% of all eligible) were enrolled in the MVUrban trial. Of these, 651 children (93% of inclusions) were enrolled in the MVPneumo study. At age 6.5 and 9 months, 515 (79%) and 529 (81%) children were followed up with a NPS, respectively (see Fig 1. **Flowchart of the study**). Specimens from 512 children having a successful swab available from both inclusion and 9 months follow up were analyzed (17 samples were lost). Of these, 410 children (80%) also had the 6.5 months swab available and analyzed. Children lost to follow-up differed on a number of factors, e.g. they were older and came from more deprived groups (data not shown). Losses to follow-up were comparable in the early MV group (20% at 6 months, 18% at 9 months) and the no early MV group (23% at 6 months, 20% at 9 months).

Of the 512 children analyzed, 346 (68%) were randomized to early MV and 166 (32%) to no early MV. Enrolment in rainy season (Jun-Nov) comprised 65% of children, and 52% of children received OPV during national campaigns before enrolment (Table 1); no children received campaign vaccines between enrolment and age 9 months. Randomization resulted in comparable groups according to all background factors (Table 1).

**Table 1:** Comparison of baseline characteristics of the two randomization groups.

Investigated variables, N (% of total number)	% (No.)		P
	Early MV	No early MV	
	N = 346 (68%)	N = 166 (32%)	
**Demographic factors**			
Age at inclusion (days), median (IQR)	145 (140–158)	147 (140–158)	
Boys, N = 281 (55)	55% (189)	55% (92)	
Girls, N = 231 (45)	45% (157)	45% (74)	0.87
**District**			
Bandim, N = 214 (42)	41% (143)	43% (71)	
Belem, N = 74 (14)	14% (49)	15% (25)	
Cuntum, N = 224 (44)	45% (154)	42% (70)	0.88
**Ethnicity**			
Papel, N = 144 (28)	29% (102)	25% (42)	
Fula, N = 81 (16)	15% (51)	18% (30)	
Manjaco, N = 68 (13)	14% (48)	12% (20)	
Other ethnicities, N = 219 (43)	42% (145)	45% (74)	0.58
**Socioeconomic status**			
The mother is literate, N = 370 (72)	72% (248)	73% (122)	
The mother is illiterate, N = 142 (28)	28% (98)	27% (44)	0.67
**Civil status**			
Unmarried mother, N = 200 (39)	39% (135)	39% (65)	
Mother married, N = 308 (60)	60% (208)	60% (100)	
Mother divorced/widow, N = 4 (1)	1% (3)	1% (1)	0.95
**The household has**			
Refrigerator, N = 124 (24)	22% (77)	28% (47)	0.13
Television, N = 246 (48)	49% (171)	45% (75)	0.37
Piped water, N = 140 (27)	25% (88)	31% (52)	0.16
Pigs in the household, N = 177 (35)	36% (123)	32% (54)	0.50
**Anthropometric values**			
Mean (SD) height (cm)	62.9 (2.7)	63.1 (2.9)	0.32
Mean (SD) weight (kg)	7.2 (1.00)	7.2 (1.01)	0.92
Mean (SD) mid upper arm circumference (mm) [NA = 2]	142 (12)	141 (12)	0.54
**Health status of the child**			
Ill at enrolment, N = 40 (8)	8% (29)	7% (11)	0.49
Mean (SD) temperature (°C) [NA = 2]	(0.4)	36.6 (0.4)	
Normal lung auscultation, N = 475 (93)	92% (319)	94% (156)	0.47
Bronchitis, N = 20 (4)	4% (14)	4% (6)	0.81
Pneumonia, N = 16 (3)	3% (12)	2% (4)	0.52
Wheezing, ever, N = 123 (24)	25% (88)	21% (35)	0.28
Coughing at enrolment, N = 208 (41) [NA = 109]	40% (139)	41%(69)	0.76
Coughing within the last 4 weeks, N = 336 (66)	65% (224)	67% (112)	0.54
Visible nasal secretions, N = 31 (6) [NA = 31]	7% (24)	4% (7)	0.32
Diarrhea, N = 51 (10)	10% (33)	11% (18)	0.64
Child is breastfed, N = 505 (99)	99% (343)	98% (162)	0.16
Mother has history of measles infection, [NA = 4] – Yes, N = 111 (22) – No, N = 220 (43) – Do not know, N = 177 (35)	22% (76) 43% (150) 34% (116)	21% (35) 42% (70) 37% (61)	0.82
Number of prior hospital admissions, median (IQR)	1 (1–1)	1 (1–1)	
Hospitalized at all before inclusion, N = 27 (5)	6% (20)	4% (7)	0.46
Child medicated within the past 3 days, N = 107 (21)	21% (74)	20% (33)	0.69
Child medicated with antibiotics within the past 3 days, N = 50 (10) [NA = 1]	10% (35)	9% (15)	0.73
**Vaccination history**			
Participated in OPV campaign prior to enrolment, N = 266 (52)	50% (172)	57% (94)	0.14
Has a BCG scar, N = 472 (92)	92% (319)	92% (153)	0.99
**Known risk factors for pneumonia/pneumococcal carriage**^a^			
No of people/bed incl. the child, median (IQR) [NA = 1]	3 (2–3)	3 (2–3)	
No of people/room incl. the child, median (IQR)	4 (3–5)	4 (3–5)	
Child is breastfed by a mother treated with antibiotics within the past week, N = 9 (2) [NA = 4]	1% (4)	3% (5)	0.31
Enrolled in rainy season (June 1 –Nov 30), N = 331 (65) Enrolled in dry season, (Dec1 –May 31), N = 181 (35)	65% (226) 35% (120)	63% (105) 37% (61)	0.65
The child is exposed to indoor fire smoke, N = 51 (10) [NA = 16]^b^	11% (38)	8% (14)	0.37
**Pneumococcal colonization/density**			
Colonized with pneumococci, N = 411 (80%)	80% (278)	80% (133)	0.95
Pneumococcal density, mean (SD)	47145 (143770)	66760 (226327)	0.24
Geometric mean = 3968	3699	4596	0.48
Geometric mean ratio (95% CI)^c^	0.80 (0.45–1.45)	1.00 (ref)	

^a^ one single parent reported cigarette smoking;

^b^information on indoor fire smoke was obtained from self-reports at 9 months of age, however, we assume that no changes in behavior related to heating/cooking has taken place since enrolment;

^c^ raw unadjusted mean ratio.

At enrolment, 80% of children were colonized with pneumococci (411/512, Table 1). At age 6.5 and 9 months, 87% and 85% of children were colonized, respectively. Using Pearson’s test of correlation, log-transformed density values were uncorrelated between inclusion and 6.5 months (ρ = 0.09, p = 0.10), whereas some correlation was found between inclusion and 9 months (ρ = 0.18, p<0.01).

Three health professionals obtained NPSs; controlling for investigator did not impact the assessment of early MV’s effect on colonization status or density (data not shown).

### Effect of early MV on pneumococcal colonization and density

The prevalence of pneumococci was similar between early MV-children and controls at age 6.5 and 9 months, PR_6.5_ 1.02 (0.94–1.10) and PR_9_ 1.04 (0.96–1.12), respectively (Table 2). No sex-differential effect was observed.

**Table 2:** Association of early measles vaccine (MV) and pneumococcal colonization and density.

	Pneumococcal colonization			Pneumococcal density	
	Early MV	No early MV	PR^a^ of colonization	Geometric mean	GMR^a^ (95% CI)
6.5 MONTHS	N colonized/N	N colonized/N	(95% CI)	Early MV/no early MV	Early MV/no early MV
Missing	NA = [70]	NA = [32]			
Overall; N = 410	241/276 (87%)	115/134 (86%)	1.02 (0.94–1.10)	2885/2820	1.02 (0.55–1.89)
Boys; N = 225	132/153 (86%)	60/72 (83%)	1.04 (0.92–1.17)	2338/2876	0.81 (0.34–1.94)
Girls; N = 185	109/123 (89%)	55/62 (89%)	1.00 (0.90–1.11)	3721/2760	1.35 (0.56–3.22)
**9 MONTHS**					
Overall; N = 512	298/346 (86%)	138/166 (83%)	1.04 (0.96–1.12)	2117/3078	0.69 (0.39–1.21)
Boys; N = 281	161/189 (85%)	77/92 (84%)	1.02 (0.91–1.13)	1727/3017	0.57 (0.27–1.23)
Girls; N = 231	137/157 (87%)	61/74 (82%)	1.06 (0.94–1.20)	2691/3157	0.85 (0.37–1.95)

^a^ Early MV versus no early MV.

No effect of MV was found on pneumococcal density at 6.5 months overall, GMR_6.5_ 1.02 (0.55–1.89). At age 9 months, the overall GMR_9_ was 0.69 (0.39–1.21) (Table 2). Again, no sex-differential effect was observed.

### Recent antibiotic treatment

At both follow up visits (Table 3) early MV-children were less frequently treated with antibiotics within the last three days compared with controls, PR_6.5_ being 0.60 (0.34–1.05) and PR_9_ 0.87 (0.50–1.53).

**Table 3:** Association between early measles vaccine (MV) and subsequent antibiotic treatment.

	Early MV	No early MV	PR of antibiotic treatment in children who
	Recently treated	Recently treated	received early MV vs. no early MV (95% CI)
**6.5 MONTHS (N = 512)**	**7%**	**12%**	0.61 (0.35–1.05)
Recently treated (N = 45)	(25/346 [NA = 53])	(20/166 [NA = 24])	
**9 MONTHS (N = 512)**	**9%**	**10%**	0.87 (0.50–1.53)
Recently treated (N = 48)	(31/346 [NA = 4])	(17/166 [NA = 2])	

NA = missing information on recent intake of antibiotics

Overall, recent antibiotic treatment was associated with lower colonization rates, PR_6.5_ 0.85 (0.71–1.01) and PR_9_ 0.66 (0.52–0.84), and decreased density, GMR_6.5_ 0.32 (0.12–0.86) and GMR_9_ 0.52 (0.18–1.52) (Table 4). Predominantly, β-lactam antibiotics such as Amoxicillin, Ampicillin, and Augmentin were used.

**Table 4:** Association between recent antibiotic treatment and pneumococcal colonization and density.

	Pneumococcal colonization			Pneumococcal density	
	Recently treated	Not recently treated	PR of colonization	Treated/not treated	
6.5 MONTHS	Colonized/N	Colonized/N	(95% CI)	Geometric mean	GMR (95% CI)
Overall; N = 512	73%	82%			
[NA = 102]*	(33/45 [NA = 1])	(320/390 [NA = 28])	0.85 (0.71–1.01)	1022/3201	0.32 (0.12–0.85)
**9 MONTHS**					
Overall; N = 512	58%	88%			
[NA = 0]**	(28/48)	(403/458)	0.66 (0.52–0.84)	1301/2530	0.51 (0.18–1.49)

NA = missing information on pneumococcal status;

*additionally, 76 children miss information on antibiotic treatment at age 6.5 months;

**additionally, 6 children miss information on antibiotic treatment at age 9 months.

### Stratified analyses: Receipt of oral polio vaccine and season

Stratification by receipt vs. no receipt of OPV during campaigns before enrolment showed no effect regarding colonization prevalence (S1 Table). At age 6.5 months, pneumococcal density tended to be reduced among early MV-children who had not participated in OPV campaigns prior to enrolment (GMR_6.5 NO OPV_ 0.50 (0.21–1.23), whereas the opposite tendency was seen among early MV-children who did receive campaign OPV before enrolment, (GMR_6.5 OPV_ 1.83 (0.78–4.28). Hence, there was a significant interaction between early MV and campaign OPV before enrolment (p = 0.04). This association was not maintained at age 9 months (GMR_9 NO OPV_ = 0.58 (0.26–1.30), GMR_9 OPV_ = 0.83 (0.38–1.82), test for interaction p = 0.53), possibly due to the effect of later OPV campaigns, as subsequent OPV campaigns tended to blur the difference between those who had and had not received campaign OPV before enrolment (data not shown).

The analysis by season of enrolment did not show any significant differences between children enrolled in the rainy and dry season, respectively (S2 Table).

## Discussion

We found no overall effect of early MV on pneumococcal colonization or pneumococcal density at age 6.5 or 9 months.

### Strengths and limitations

The randomization ensured that background variables were balanced between randomization groups (Table 1).

#### Post randomization imbalances—Antibiotic treatment

In post hoc analyses we found that early MV-children were less frequently treated with antibiotics at follow up. This is in line with the findings of a morbidity subgroup study within the main trial, which found that early MV was associated with a reduced risk of morbidity from 4 to 9 months of age[5]. Thus, as anticipated, early MV may indeed have had beneficial non-specific effects on overall health. This, however, could have large implications for the outcome of the present trial, because recent antibiotic treatment was associated with fewer colonized children and lower pneumococcal density. The association between antibiotic treatment and pneumococcal colonization and density is well-known; transient reducing effects on overall pneumococcal colonization have been shown for β-lactam antibiotics [19, 20] and macrolides [21] when measured by culture, as well as for antimicrobials in general when measured with PCR [22, 23]. Thus, a protective effect of MV on pneumococcal colonization could have been masked by antibiotic treatment being overrepresented among controls.

However, we chose not to adjust for antibiotic treatment in the analyses, because bias would be introduced when adjusting or stratifying for a post randomization variable associated with a prior intervention[24] (so-called *inconsistent mediation*[25]).

Unfortunately, antibiotic treatment of children was only registered if taken within three days prior to examination so we are unsure if this observation reflects differences in the overall consumption of antibiotics in each group. Also, no objective measure of antibiotic activity in a child was assessed. Information on antibiotic intake relied solely on reporting by mothers. They do not necessarily know whether a given medication prescribed by the doctor is an antibiotic and they might not remember the exact name of the medicine. At the 6.5 months visit, however, the information was also based on seeing the medicine packaging by the home visiting nurse, increasing the reliability of the information at this visit.

#### Sample size and loss to follow up

The sample size was intended to include minimum 420 early MV-children and 210 controls. However, only samples from 346 and 166 children (early MV/no early MV) were analyzed resulting in a statistical power to show a significant difference of 74% in contrast to the anticipated power of 80%.

Additionally, 21% and 19% of enrolled children were lost to follow up at 6.5 and 9 months of age, respectively, and comprised a predominance of the less advantaged children (data not shown), which may reduce the generalizability of the findings. However, since loss to follow-up was evenly distributed between randomization groups we do not believe it affected the estimated early MV effect.

### Consistency with other studies

#### Pneumococcal carriage rates

Pneumococcal carriage rates among infants in Guinea-Bissau have not previously been investigated, but the observed rates are consistent with reports from other West African countries: The Gambia[11] (80% prevalence by the age of 13 weeks), Burkina Faso[26] (73% among 6–11 months old), and Nigeria[27] (approximately 90% among 6–9 months old), before the introduction of PCV, which took place in 2009, 2013, and 2014, respectively[28].

#### Effect of yellow fever vaccine and oral polio vaccine

In the Gambian before-after study [7] a 75% reduction of NP pneumococcal carriage (OR 0.25 (0.07–0.90)) was seen after co-administration of measles and yellow fever vaccine at age 9 months. The effect was strongest within 4 weeks following vaccination, and non-significant >4 weeks after. In the present study, at least 2 months passed from enrolment to first follow up because we aimed at exploring longer-term effects so a transient effect of MV might have been missed.

Additionally, the YF vaccine could have beneficial NSEs like other live vaccines such as MV, BCG[29], smallpox[30], and OPV[31–33]. In the present study, we investigated the effect of early MV alone since YF vaccine is only recommended at age 9 months.

Two national OPV campaigns were conducted within the study period (Table 1). Data from a previous trial suggest that receiving OPV in campaigns prior to early MV is associated with a less beneficial effect of early MV[18]. We saw the same tendency in the present study (S1 Table), with a more beneficial effect of early MV on pneumococcal density in children who had not received campaign-OPV before enrolment.

Our data did not support a stronger beneficial effect of early MV among children enrolled in dry season as indicated by a previous Guinean trial[4] (S2 Table).

### Interpretation

Previous Danish and Guinean studies have observed beneficial NSEs of MV on respiratory morbidity[4, 6]. Supported by the Gambian before-after study we hypothesized that this could be mediated via an effect on pneumococcal colonization. This hypothesis was not confirmed. There are several potential explanations for that.

First, preponderance of antibiotic treatment in the control group may have masked an effect of early MV on pneumococcal colonization along with a possibly blurring effect of the national OPV-campaigns. WHO has called for more studies into non-specific effects of vaccines, and particularly encouraged more RCTs[34]. The present study is a good example of the potential caveats in conducting RCTs. In real life, if the intervention changes their post-randomization health, children in a randomized trials can only be considered “randomized” on the day of enrolment. The present study indicated that early MV-children may have had less antibiotics (i.e. a beneficial non-specific effect of early MV). This again may have affected the outcome of the study, pneumococcal colonization. Importantly, if one tries to adjust for such post-randomization imbalances additional bias is potentially introduced. Furthermore, there are now many indications that health interventions interact [35, 36], and thus interventions occurring both before and after the RCT intervention, e.g., OPV campaigns, may blur the effect of the RCT intervention in unforeseen ways. This calls for more advanced epidemiological tools to evaluate the real-life effect of health interventions in the context of other interventions.

Secondly, viruses are also important causes of respiratory infections in the first years of life [37], e.g. RSV[38] and influenza virus [39]. Indeed, MV has been associated with a reduced rate of hospital contacts with laboratory-confirmed RSV infections in Denmark [40]. Viral infections paving the way for invasive bacterial infection is well-documented [41, 42], including promotion of pneumococci by influenza virus.[43, 44] Hence, MV may indirectly decrease bacterial co-/superinfection by inducing immunity against heterologous viruses rather than changing the NP bacterial colonization per se.

Moreover, the nasopharynx is dynamic, with several potentially disease-causing microorganisms constituting a commensal ecosystem with competition and promotion species in-between. Hence, more comprehensive mapping of the entire microbial flora and more frequent swabbing immediately following MV would strengthen the investigation of NSEs on microbial carriage.

In conclusion, the present study cannot confirm or refute previous observations of a beneficial effect of MV on pneumococcal colonization nor on density.

The association of MV and reduced respiratory morbidity reported in previous studies calls for further investigations with early and frequent sampling allowing for detailed surveys of pathogens causing respiratory disease in early childhood, including both viruses and bacteria. The present study may serve as a baseline for future evaluation of the impact of PCV-13 that was introduced in Guinea-Bissau in 2015.

## Supporting information

S1 Appendix Detection of *S*. *Pneumoniae* through quantitative Real-Time PCR. (DOCX)

S1 Table Effect of early measles vaccine (MV) on pneumococcal colonization and density at 6.5 and 9 months of age stratified by receipt of oral polio vaccine (OPV) in national vaccination campaigns prior to enrolment. (DOCX)

S2 Table Effect of early measles vaccine (MV) on pneumococcal colonization and density at 6.5 and 9 months of age stratified for season by enrolment. (DOCX)

S1 Checklist CONSORT 2010 checklist of information to include when reporting a randomised trial. (DOC)

S1 Protocol Protocol in English. Effect of early measles vaccine on pneumococcal colonization: A randomized trial from Guinea-Bissau.
(DOC)

S2 Protocol Protocol in Portuguese. Estudo dos Efeitos não Específicos da Vacina do Sarampo na presença de S.pneumoniae nas vias aéreas nasais das Crianças Guineenses.
(DOCX)
