# Correction: Effect of early measles vaccine on pneumococcal colonization: A randomized trial from Guinea-Bissau

**DOI:** 10.1371/journal.pone.0224782

**Published:** 2019-10-30

**Authors:** Nadja Skadkær Hansen, Stine Byberg, Lars Hervig Jacobsen, Morten Bjerregaard-Andersen, Aksel Karl Georg Jensen, Cesario Martins, Peter Aaby, Jørgen Skov Jensen, Christine Stabell Benn, Hilton Whittle

The ninth author's initials are indexed incorrectly in PubMed. The correct initials are: Benn CS.
