# Supplementary material for: Effect of early measles vaccine on pneumococcal colonization: A randomized trial from Guinea-Bissau
Source: PLoS One. 2017 May 17;12(5):e0177547. doi: 10.1371/journal.pone.0177547 (PMC5435222; doi:10.1371/journal.pone.0177547)
Supplement: S1 Appendix — (DOCX) [file pone.0177547.s001.docx]

**S1 Appendix**

**Detection of *S. Pneumoniae* through quantitative Real-Time PCR**

Genomic DNA was extracted from 125 µl of the NPS specimen solution using the FastDNA^TM^ spin kit for soil (MPBiomedicals, LLC, Santa Ana, CA, USA) with elution into 100 µl. The number of pneumococci detected from 0.25 µl DNA-extraction was determined using a q-PCR detecting a specific segment of the autolysin gene (*lyt*A) with primers lytA-F373 ACGCAATCTAGCAGATGAAGCA and lytA-R424 TCGTGCGTTTTAATTCCAGCT and the probe lytA-Pb400taqman FAM-TGCCGAAAACGCTTGATACAGGGAG-BHQ1 as described by the US CDC^1^ with a slight modification of the forward primer and the probe. Furthermore, an internal amplification control was constructed from phage lambda DNA as previously described^2^ and detected with the probe Lambda IPC taqman rev TAMRA-TCCTTCGTGATATCGGACGTTGGCTG-BHQ2. PCR was performed in a 50 μl final volume with 1x PerfeCTa™ SuperMix with UNG and low ROX (Quanta Biosciences, INC., Gaithersburg, MD, USA), 320 nM forward and reverse primers, and 75 nM probe. Thermocycling was conducted on an ABI 7500 real-time PCR instrument programmed for an initial denaturation for 2 min at 95°C followed by 50 cycles each consisting of a 95°C, 15 sec. denaturation and a 60°C 1 min annealing and extension. A standard curve was constructed from 10-fold dilutions of purified *S. Pneumoniae* DNA in TE buffer containing calf-thymus DNA as stabilizer/carrier DNA. Results were analysed using 7500 Software v2.3, Applied Biosystems. In case of absence of internal control the sample was re-analysed. All analyses were conducted at Statens Serum Institut in Denmark.
