# Supplementary material for: Effect of early measles vaccine on pneumococcal colonization: A randomized trial from Guinea-Bissau
Source: PLoS One. 2017 May 17;12(5):e0177547. doi: 10.1371/journal.pone.0177547 (PMC5435222; doi:10.1371/journal.pone.0177547)
Supplement: S1 Table — (DOCX) [file pone.0177547.s002.docx]

**S1 Table.**

**Effect of early measles vaccine (MV) on pneumococcal colonization and density at 6.5 and 9 months of age stratified by receipt of oral polio vaccine (OPV) in national vaccination campaigns prior to enrolment.**

|  |  | **PR** of colonization (95% CI) | | | **GMR** (95% CI) | | |
| --- | --- | --- | --- | --- | --- | --- | --- |
|  | N colonized/N (%) OPV/no OPV | Campaign-OPV | No campaign OPV | Test for interaction | Campaign- OPV | No campaign-OPV | Test for interaction |
| 6.5 MONTHS |  | N=217 [NA=48] | N= 193 [NA=54] |  | N=190 | N=166 |  |
| **Overall** | 190/166 (88/86) | 1.02 (0.94-1.10) | 1.00 (ref.) |  | 0.91 (0.51-1.62) | 1.00 (ref.) |  |
| Early MV | 126/115 (88/87) | 1.00 (0.90-1.11) | 1.04 (0.92-1.19) |  | 1.83 (0.78-4.28) | 0.50 (0.21-1.23) |  |
| Controls | 64/51 (88/84) | 1.00 (ref.) | 1.00 (ref.) | P = 0.61 | 1.00 (ref.) | 1.00 (ref.) | P = 0.04 |
| **Boys** | 100/92 (85/86) | 0.99 (0.88-1.10) | 1.00 (ref.) |  | 0.76 (0.34-1.70) | 1.00 (ref.) |  |
| Early MV | 68/64 (86/86) | 1.05 (0.88-1.25) | 1.02 (0.86-1.21) |  | 1.58 (0.46-5.40) | 0.38 (0.11-1.32) |  |
| Controls | 32/28 (82/85) | 1.00 (ref.) | 1.00 (ref.) | P = 0.82 | 1.00 (ref.) | 1.00 (ref.) | P = 0.11 |
| **Girls** | 90/74 (91/86) | 1.06 (0.95-1.17) | 1.00 (ref.) |  | 1.11 (0.49-2.54) | 1.00 (ref.) |  |
| Early MV | 58/51 (89/88) | 0.95 (0.84-1.07) | 1.07 (0.88-1.31) |  | 2.22 (0.68-7.28) | 0.71 (0.19-2.64) |  |
| Controls | 32/25 (94/82) | 1.00 (ref.) | 1.00 (ref.) | P = 0.30 | 1.00 (ref.) | 1.00 (ref.) | P = 0.20 |
|  |  |  |  |  |  |  |  |
| 9 MONTHS |  | N=265 | N=247 |  | N=221 | N=215 |  |
| **Overall** | 221/215 (83/87) | 0.96 (0.89-1.03) | 1.00 (ref.) |  | 1.37 (0.81-2.31) | 1.00 (ref.) |  |
| Early MV | 145/153 (84/88) | 1.03 (0.92-1.16) | 1.04 (0.93-1.16) |  | 0.83 (0.38-1.82) | 0.58 (0.26-1.30) |  |
| Controls | 76/62 (82/85) | 1.00 (ref.) | 1.00 (ref.) | P = 0.97 | 1.00 (ref.) | 1.00 (ref.) | P = 0.53 |
| **Boys** | 118/120 (81/88) | 0.92 (0.84-1.02) | 1.00 (ref.) |  | 0.76 (0.34-1.70) | 1.00 (ref.) |  |
| Early MV | 77/84 (83/88) | 1.05 (0.89-1.24) | 0.97 (0.85-1.11) |  | 0.54 (0.18-1.63) | 0.63 (0.21-1.88) |  |
| Controls | 41/36 (79/90) | 1.00 (ref.) | 1.00 (ref.) | P = 0.48 | 1.00 (ref.) | 1.00 (ref.) | P = 0.85 |
| **Girls** | 103/95 (86/86) | 1.00 (0.90-1.11) | 1.00 (ref.) |  | 1.23 (0.60-2.64) | 1.00 (ref.) |  |
| Early MV | 68/69 (86/88) | 1.01 (0.86-1.18) | 1.12 (0.92-1.36) |  | 1.35 (0.43-4.24) | 0.50 (0.15-1.72) |  |
| Controls | 35/26 (85/79) | 1.00 (ref.) | 1.00 (ref.) | P = 0.40 | 1.00 (ref.) | 1.00 (ref.) | P = 0.25 |
