# Supplementary material for: Effect of early measles vaccine on pneumococcal colonization: A randomized trial from Guinea-Bissau
Source: PLoS One. 2017 May 17;12(5):e0177547. doi: 10.1371/journal.pone.0177547 (PMC5435222; doi:10.1371/journal.pone.0177547)
Supplement: S2 Table — (DOCX) [file pone.0177547.s003.docx]

**S2 Table.**

**Effect of early measles vaccine (MV) on pneumococcal colonization and density at 6.5 and 9 months of age stratified by season at enrolment.**

|  |  | **PR** of colonization (95% CI) | | | **GMR** (95% CI) | | |
| --- | --- | --- | --- | --- | --- | --- | --- |
|  | N colonized/N (%) rainy/dry season | Enrolled in rainy season | Enrolled in dry season | Test for interaction | Enrolled in rainy season | Enrolled in dry season | Test for interaction |
| 6.5 MONTHS |  | N=264 [NA=67] | N=146 [NA=35] |  | N=229 | N=127 |  |
| **Overall** | 229/127 (87/87) | 1.00 (0.92-1.08) | 1.00 (ref.) |  | 1.31 (0.72-2.38) | 1.00 (ref.) |  |
| Early MV | 154/87 (86/91) | 0.96 (0.87-1.05) | 1.13 (0.97-1.32) |  | 0.70 (0.33-1.48) | 2.08 (0.72-6.02) |  |
| Controls | 75/40 (89/80) | 1.00 (ref.) | 1.00 (ref.) | P = 0.07 | 1.00 (ref.) | 1.00 (ref.) | P = 0.10 |
| **Boys** | 127/65 (84/88) | 0.96 (0.86-1.07) | 1.00 (ref.) |  | 1.01 (0.43-2.37) | 1.00 (ref.) |  |
| Early MV | 82/50 (82/94) | 0.93 (0.81-1.07) | 1.32 (1.00-1.75) |  | 0.80 (0.28-2.32) | 0.84 (0.17-4.20) |  |
| Controls | 45/15 (88/71) | 1.00 (ref.) | 1.00 (ref.) | P = 0.03 | 1.00 (ref.) | 1.00 (ref.) | P = 0.10 |
| **Girls** | 102/62 (90/86) | 1.05 (0.94-1.17) | 1.00 (ref.) |  | 1.79 (0.80-4.17) | 1.00 (ref.) |  |
| Early MV | 72/37 (90/86) | 0.99 (0.87-1.13) | 1.00 (0.83-1.21) |  | 0.53 (0.18-1.54) | 4.33 (0.98-19.09) |  |
| Controls | 30/25 (91/86) | 1.00 (ref.) | 1.00 (ref.) | P = 0.94 | 1.00 (ref.) | 1.00 (ref.) | P = 0.02 |
|  |  |  |  |  |  |  |  |
| 9 MONTHS |  | N=331 | N=181 |  | N=287 | N=149 |  |
| **Overall** | 287/149 (87/82) | 1.05 (0.97-1.14) | 1.00 (ref.) |  | 0.87 (0.50-1.51) | 1.00 (ref.) |  |
| Early MV | 197/101 (87/84) | 1.02 (0.93-1.12) | 1.07 (0.92-1.25) |  | 0.82 (0.41-1.66) | 0.49 (0.19-1.27) |  |
| Controls | 90/48 (86/79) | 1.00 (ref.) | 1.00 (ref.) | P = 0.58 | 1.00 (ref.) | 1.00 (ref.) | P = 0.40 |
| **Boys** | 160/78 (85/85) | 1.00 (0.90-1.11) | 1.00 (ref.) |  | 1.33 (0.62-2.86) | 1.00 (ref.) |  |
| Early MV | 107/54 (85/86) | 1.01 (0.89-1.15) | 1.04 (0.85-1.26) |  | 0.75 (0.29-1.98) | 0.32 (0.09-1.17) |  |
| Controls | 53/24 (84/83) | 1.00 (ref.) | 1.00 (ref.) | P = 0.83 | 1.00 (ref.) | 1.00 (ref.) | P = 0.31 |
| **Girls** | 127/71 (89/80) | 1.12 (1.00-1.26) | 1.00 (ref.) |  | 0.55 (0.25-1.21) | 1.00 (ref.) |  |
| Early MV | 90/47 (90/82) | 1.02 (0.90-1.16) | 1.10 (0.87-1.39) |  | 0.92 (0.32-2.61) | 0.82 (0.20-3.29) |  |
| Controls | 37/24 (88/75) | 1.00 (ref.) | 1.00 (ref.) | P = 0.59 | 1.00 (ref.) | 1.00 (ref.) | P = 0.90 |
