# Supplementary material for: Effect of early measles vaccine on pneumococcal colonization: A randomized trial from Guinea-Bissau
Source: PLoS One. 2017 May 17;12(5):e0177547. doi: 10.1371/journal.pone.0177547 (PMC5435222; doi:10.1371/journal.pone.0177547)
Supplement: S1 Protocol — Effect of early measles vaccine on pneumococcal colonization: A randomized trial from Guinea-Bissau. (DOC) [file pone.0177547.s005.doc]

**S1. Protocol in English**

**Effect of early measles vaccine on pneumococcal colonization: A randomized trial from Guinea-Bissau**

**Summary**

Recent studies from Guinea-Bissau and Denmark have shown that measles vaccine (or measles-mumps-rubella vaccine in Denmark) protects children against lower respiratory infections.

*Streptococcus pneumoniae* is a leading cause of lower respiratory infections. In the present study we investigate effect of providing an early MV at age 4.5 months on the risk of nasopharyngeal colonization with *S. pneumoniae* in children in Guinea-Bissau. We hypothesize that early MV reduces the carriage rate from 85% to 75%. Within a randomized clinical trial of early MV we will enroll 420 children who receive an early MV and 210 controls who receive no early MV. All children will have a nasopharyngeal swab performed immediately before randomization at 4.5 months of age and eight weeks after, as well as by 9 months of age, immediately before the recommended MV. Colonization with *S. pneumoniae* will be determined by quantitative PCR.

**Background**

Many observational studies in different countries^1,2^ and a randomized trial in Guinea-Bissau^3^ have shown beneficial effects of measles vaccine (MV) on child survival which are independent of its direct effect of preventing measles. In the randomized trial the mortality rate ratio (MRR) of children given MV at 4.5 and 9 months of age compared to those given a single dose at 9 months of age was 0.70 (0.52-0.94), independently of the protective effect on measles infection^3^. Analysis of a subgroup of children showed that children with maternal measles antibody at the time of the first MV had a markedly enhanced survival rate (MRR 0.17; 0.05-0.58) compared to children given the first MV when maternal measles antibody was absent (Aaby et al, submitted).

Analysis of hospital admissions showed a reduction in early MV group, which was strongest for pneumonia/respiratory infections; the admissions hazard ratio was 0.37 (0.37; 0.16-0.89) compared with the controls (Aaby et al, submitted). This finding was recently supported in a register-based study among Danish children, in which measles-mumps-rubella vaccination was associated with a 21% (95% CI 17-24%) reduction in the risk of lower respiratory infections (Sørup et al, submitted). It should be noted that these findings were done in situations where

1

there was no circulating measles virus and thus were unrelated to the measles-preventive effect of the vaccine.

Preliminary studies in The Gambia suggest that measles vaccine may reduce nasopharyngeal carriage of *S. pneumoniae*. These observations fit with strong evidence from animal studies that priming with one pathogen may induce heterologous immunity and thus reduce susceptibility to subsequent infection with another unrelated pathogen^4,5^.

Globally, *S. pneumoniae* infections are a leading cause of pediatric morbidity and mortality; it is estimated that *S. pneumoniae* is responsible for more than 800,000 deaths and nearly 15 million severe infections, including pneumonia, annually in children below 5 years of age, with 90% of cases occurring in low-income countries^6^. Asymptomatic nasopharyngeal colonization is considered to be the first step in the pathway to infection^7^. Children in low-income settings experience early and abundant *S. pneumoniae* carriage very early in life^8^. Pneumococcal conjugate vaccines prevent invasive infections, but have not yet been implemented in many poor countries, including Guinea-Bissau.

At the Bandim Health Project in Guinea-Bissau, a large randomized trial examining the impact of maternal antibody on the non-specific effects of measles vaccine is in progress. Normally children receive MV at 9 months of age. In the trial infants are randomized 2:1 to two doses of MV at 4.5 and 9 months of age or a single MV at 9 months of age. All children have blood collected at enrolment for assessment of maternal antibody levels. Mortality and the frequency of hospital admissions will be compared between the groups overall and within the periods 4-8 months and 9-36 months. The data will be analyzed according to presence of maternal measles antibody at 4.5 months to test the hypothesis that early MV is particularly beneficial when given in the presence of maternal antibodies.

We propose that one of the mechanisms by which early MV exerts its beneficial non-specific effects on mortality and the risk of hospital admissions for respiratory infections is by reducing the carriage of *S. pneumoniae*. Thus, within the main trial we have designed a sub study to test the hypothesis that early MV reduced the risk of being colonized with *S. pneumoniae*.

2

**Methods**

In the capital Bissau, the Bandim Health Project (BHP) is running a health and demographic surveillance system which covers more than 102,000 individuals in 6 suburbs. All residents are given a unique identification number and information on background factors including socio-economic factors is collected. All houses in the BHP study area are visited monthly by an assistant who registers new pregnancies and births. Once a newborn is identified, the child is followed with trimonthly visits until 3 years of age. Information on vital status and vaccination status is registered at each visit.

***Study design***

The present study is nested within the Early MV trial, a randomized controlled trial (RCT), which was initiated in August 2011 and will continue to enroll children until a sample size of 6600 children is reached, anticipated by the end of 2014. A subgroup of the participants in the trial will be invited to participate in the present study.

***The Early MV trial***

The primary objective of the early MV trial is to study the effect of early MV at 4.5 months versus no early MV at 4.5 months on overall mortality until 36 months of age. All children receive the recommended MV at 9 months. Inclusion criteria are that the child has received the third dose of pentavalent vaccines recommended at 14 weeks of age, at least four weeks before enrolment. Provided oral and written consent from the mother/guardian, the child is examined clinically to identify ill children in need of hospitalization, children with malformations impairing health and malnourished children (defined as a mid-upper-arm circumference (MUAC) less than 115 mm). These infants are temporary excluded, but are invited to be enrolled in the study when they have recovered.

A blood sample is obtained for determination of maternal measles antibody levels. All children who are invited to participate are offered free consultations at the local health centers and essential drugs free of charge. A questionnaire is filled out, covering the health of the child and socio-economic factors.

3

Infants are randomized by block randomization procedures, stratified by sex. Each envelope contains 24 lots, 2/3 of the lots assign the child to the intervention group (early MV), 1/3 of the lots assign the child to the control group (no early MV). Twins of the same sex are allocated to the same treatment to avoid confusion. Children randomized to early MV receive a single dose of 0.5 ml MV (Edmonston-Zagreb strain, Serum Institute of India) as a subcutaneous injection into the upper part of their back. No placebo is given, since mothers could get the false impression that their child was measles vaccinated.

***The pneumococcal sub-study***

*Objective*

In a subset of children we will test the effect of early MV on *S. pneumoniae* carriage. Thus, 420 children randomized to MV and 210 controls randomized to no MV will have a nasopharyngeal swab performed immediately before randomization, and at 6.5 and again at 9 months of age (immediately before MV) and colonization with *S. pneumoniae* will be determined (Figure 1).


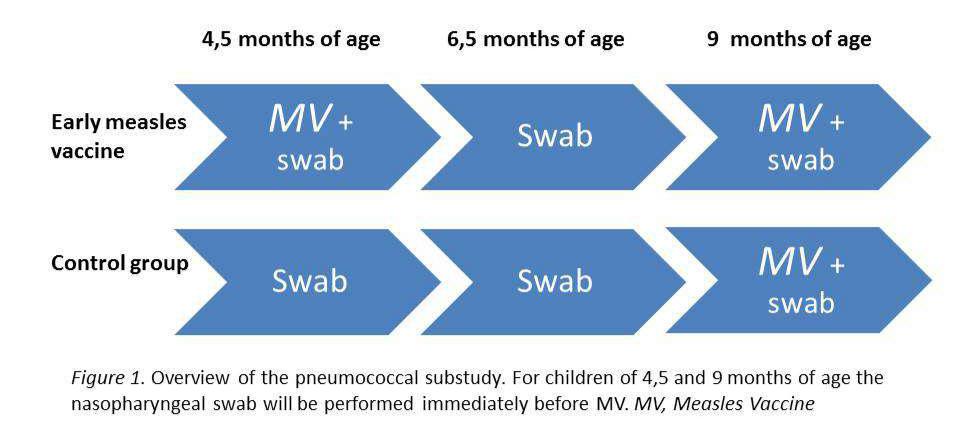


*Hypothesis*

We hypothesize that early MV at 4.5 months of age is associated with a reduction in the risk of being colonized by *S. pneumoniae* at 6.5 and 9 months of age.

4

*Nasopharyngeal swabs*

Nasopharyngeal specimens will be obtained following a set protocol. A small, flexible, swab is inserted into one of the nares to the level of the posterior nasopharynx, a distance approximately midway between the tip of the nose and the ear lobe^9,10^. Swab specimens will be

placed in a labeled vial containing 1 ml skim milk-tryptone-glucose-glycerin (STGG) medium and transported on ice to the laboratory within three hours^9^. A 0.5 ml aliquot of the sample will be mixed with a DNA/RNA stabilizing transport medium for future molecular testing. The samples will be frozen at -40°C and will be transported to Statens Serum Institut, Denmark, for further processing.

*Detection of* S. pneumoniae *by quantitative PCR*

PCR will be performed to determine the total load of pneumococci using a quantitative real-time PCR method detecting a specific segment of the autolysin gene (*lyt*A) and incorporating an internal control to monitor inhibition.

*Further studies on the collected specimens*

For the present study the quantitative PCR results will be used. Remnants of the extracted DNA and original samples will be stored at SSI. Should funding become available, we will also test it for the presence of other pathogens and for changes in the microbial flora. Furthermore, should funding become available, the sample in the STGG medium will be used for culture and serotyping of pneumococci, providing pre-vaccination baseline data on pneumococcal serotype distribution in Guinea-Bissau.

*Statistical analysis*

Analyses will be conducted on the basis of intention to treat using Stata. Baseline characteristics of the treatment groups will be compared using chi square tests for contingency data and t tests for continuous data. Multivariate Poisson models with robust variance estimation providing prevalence ratios (PR) will be used to evaluate the effect of early MV overall and stratified by potential effect modifiers: sex and presence of maternal antibodies. Differences will be considered significant at P<0.05.

5

*Sample size calculations*

The prevalence of *S. pneumoniae* carriage in Guinea-Bissau is unknown, but based on data from similar areas^12,13^ we assume that the prevalence in the control group will be 85% and with 420 children in the intervention group and 210 children in the control we will be able to show a reduction from 85% to 75% associated with early MV with a power of 80% and p<0.05. We have chosen to keep the 2:1 ratio to explore the potential role of maternal antibodies.

**Ethical considerations**

The early MV trial is approved by the Ethical Committee in Guinea-Bissau. The Danish Central Ethical Committee gave its consultative approval. The present protocol will be submitted for approval by the same committees.

**Project group**

Nadja Skadkær, Medical student, University of Copenhagen, will be scholarship student. Peter Aaby (DMSc), Christine S. Benn (MD, DMSc) and Cesario Martins (MD, PhD) are the PIs for the Early MV trial and will be supervisors. Morten Bjerregaard-Andersen (MD) is post-doc at the Research Center for Vitamins and Vaccines, but stationed in Guinea-Bissau at the Bandim Health Project. He has previously worked on bacteriological studies and will assist Nadja Skadkær in daily practical matters. Hilton Whittle designed the present study study and will be the senior supervisor. The PCR analyses will be carried out at Department for Microbiology and Infection Control at Statens Serum Institut, in collaboration with Jørgen Skov Jensen and others.

**Perspectives**

It will be important to know whether the beneficial effects of early measles vaccine on overall mortality and not least lower respiratory infections is mediated via an effect on pneumococcal colonization. Furthermore the data will provide information on *S. pneumoniae* carriage rates among infants in Guinea-Bissau. A recent study has linked carriage of *S. pneumoniae* to impaired infant growth^8^; this finding can also be tested with the present material.

6

**References**

- Aaby, P. *et al.* Non-specific beneficial effect of measles immunisation: analysis of mortality studies from developing countries. *BMJ* **311**, 481-485 (1995).
- Aaby, P. *et al.* The optimal age of measles immunisation in low-income countries: a secondary analysis of the assumptions underlying the current policy. *BMJ Open* **2**, doi:10.1136/bmjopen-2011-000761 (2012).
- Aaby, P. *et al.* Non-specific effects of standard measles vaccine at 4.5 and 9 months of age on childhood mortality: randomised controlled trial. *BMJ* **341**, c6495 (2010).
- Welsh, R. M. & Selin, L. K. No one is naive: the significance of heterologous T-cell immunity. *Nat Rev Immunol* **2**, 417-426, doi:10.1038/nri820 (2002).
- Kleinnijenhuis, J. *et al.* Bacille Calmette-Guerin induces NOD2-dependent nonspecific

protection from reinfection via epigenetic reprogramming of monocytes. *Proceedings of* *the National Academy of Sciences of the United States of America* **109**, 17537-17542, doi:10.1073/pnas.1202870109 (2012).

- O'Brien, K. L. *et al.* Burden of disease caused by Streptococcus pneumoniae in children younger than 5 years: global estimates. *Lancet* **374**, 893-902, doi:10.1016/S0140-6736(09)61204-6 (2009).
- Bogaert, D., De Groot, R. & Hermans, P. W. Streptococcus pneumoniae colonisation: the key

to pneumococcal disease. *Lancet Infect Dis* **4**, 144-154, doi:10.1016/S1473-

3099(04)00938-7 (2004).

- Coles, C. L. *et al.* Pneumococcal carriage at age 2 months is associated with growth deficits

at age 6 months among infants in South India. *J Nutr* **142**, 1088-1094,

doi:10.3945/jn.111.156844 (2012).

- O'Brien, K. L., Nohynek, H. & World Health Organization Pneumococcal Vaccine Trials

Carriage Working, G. Report from a WHO Working Group: standard method for detecting upper respiratory carriage of Streptococcus pneumoniae. *Pediatr Infect Dis J* **22**, e1-11, doi:10.1097/01.inf.0000049347.42983.77 (2003).

1. Kaltoft, M. S., Skov Sorensen, U. B., Slotved, H. C. & Konradsen, H. B. An easy method for detection of nasopharyngeal carriage of multiple Streptococcus pneumoniae serotypes. *Journal of microbiological methods* **75**, 540-544, doi:10.1016/j.mimet.2008.08.010 (2008).

7

1. Corless, C. E. *et al.* Simultaneous detection of Neisseria meningitidis, Haemophilus influenzae, and Streptococcus pneumoniae in suspected cases of meningitis and septicemia using real-time PCR. *J Clin Microbiol* **39**, 1553-1558, doi:10.1128/JCM.39.4.1553-1558.2001 (2001).
2. Roca, A. *et al.* Effects of community-wide vaccination with PCV-7 on pneumococcal nasopharyngeal carriage in the Gambia: a cluster-randomized trial. *PLoS medicine* **8**, e1001107, doi:10.1371/journal.pmed.1001107 (2011).
3. Coles, C. L. *et al.* Newborn vitamin A supplementation does not affect nasopharyngeal carriage of Streptococcus pneumoniae in Bangladeshi infants at age 3 months. *J Nutr* **141**, 1907-1911, doi:10.3945/jn.111.141622 (2011).

8


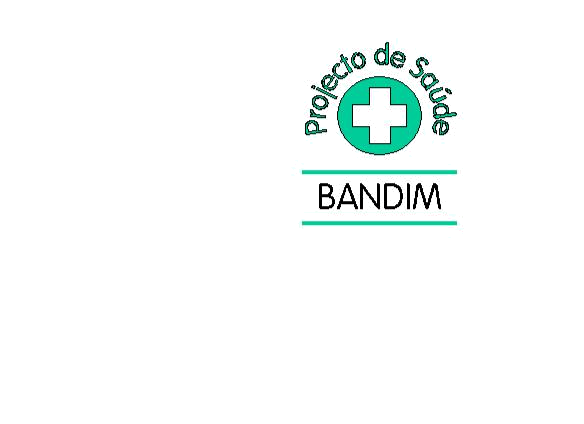


**The effect of early measles vaccine on pneumococcal colonization**

**Information letter (will be translated into Portuguese)**

**Objective**

The Bandim Health Project is investigating whether early measles vaccine can influence the risk of getting colonized in the nose with the bacteria pneumococci.

**Approaches**

Your child has been included in a study of early measles vaccine. We would like to test a subgroup of children from this study for the presence of pneumococci in the airways. Many children probably have pneumococci in the nose, and only a few of the children get ill from the bacteria, but it will be important if we can show that measles vaccine influences the risk of getting colonized and to what extent. The test involves taking a swab from the nose of your child. We would also like to measure and weigh your child and ask some questions about your child’s health.

**Benefits**

By participating in this project you will help us learn about the effects of measles vaccine and may help children in the future.

**Risks and adverse events**

The testing procedure is very safe. The test causes a slight discomfort and your child may cry, but it usually stops again quickly after. Other than that, there are no risks.

**Information**

All personal information will be kept confidential and stored securely by the Bandim Health Project. You can obtain information stored about you by request from the Bandim Health Project. Results of the study will be reported as a summary to other scientists internationally, and no individual results will be reported. Data will be retained indefinitely and may be used in other related research by the Bandim Health Project. De-identified information may be shared with other researchers in the future to use in research related to this project.

**Voluntary**

Participation in the study is voluntary. The decision to be included or not will not affect your relationship in any way with the Bandim Health Project in the future.

If you have questions or would like more information about this study, please contact Dr. Cesario Martins at the Bandim Health Project.

Projecto de Saúde de Bandim

Apertado 861, 1004 Bissau Codex, Guiné-Bissau


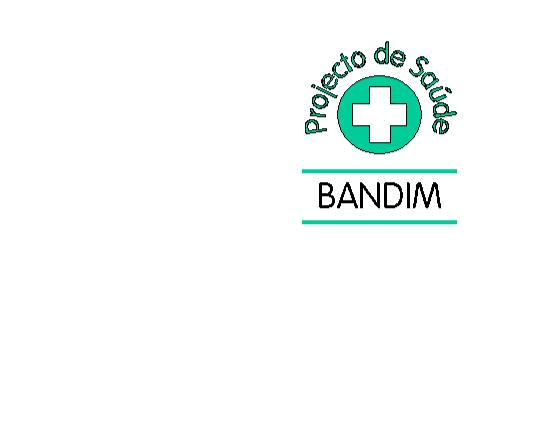


**The effect of early measles vaccine on pneumococcal colonization**

**Informed consent (will be translated into Portuguese)**

The information letter has been read out loud to me or I have read the information letter. I understand the information given. I furthermore consent that:

- The information gathered from the study is confidential.
- A nasal swab will be performed to test the child for the presence of pneumococci now, when my child is around 6.5 months old, and again when it is 9 months old, before it receives measles vaccine.
- Information obtained may be shared with other researchers in the future. If information sharing occurs, such data will be de-identified.
- Participation is based on my full consent. I can withdraw from participating in the study at any time without further explanation. To withdraw from the study will not compromise any relation between me and my child and the Bandim Health Project.

I have been given the opportunity to ask any question regarding the present study and my participation. Questions have been answered satisfactorily.

My participation is voluntary and based on my sovereign decision.

Name of the child _____________________________________________________

Name of the mother or guardian _________________________________________

Signature or fingerprint _____________________________________________

Date |__|__|-|__|__|-|2|0| _ |__|


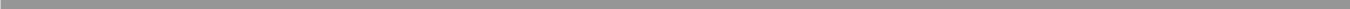


I _________________________________________________________(name),

co-worker at Bandim Health Project, declare that I have explained the study and the implications to the study participant. The participant has understood the study and its implications, and provided his/her consent to participate.

Signature ________________________________________________________

Date |__|__|-|__|__|-|2|0| _ |__|

**The effect of early measles vaccine on pneumococcal colonization**

**Inclusion questionnaire**

**General information:**

Date of inclusion: __/___/____ Study identification number:___________________

Child’s name:____________________________________________ Sex:___ (1: Male, 2: Female) Child’s date of birth:___/_____/_____

Mother/guardian name: ________________________________ Ethnic group:________________

Telephone number:_____________________

Relation to child:___ (1: Mother, 2: Father, 3: Aunt, 4: Grandmother, 5: Other) Accepts to participate in the study: (1:Yes, 2: No)

**Socio-economic status: (Most information contained in the inclusion questionnaire to the main trial)**

Mother can read/write:___ (1: Yes, 2: No, 3: Unknown)

Marital status:____(1: Single, 2: Married, 3: Divorced, 4: Unknown)

Maternal schooling: ___ (1: Primary, 2: Secondary, 3: High school, 4: Univers., 5: No edu. , 6: Unknown)

Household has:

Water tap inside: (1: Yes, 2: No, 3:Unknown) Television:___(1: Yes, 2: No, 3: Unknown) Refrigerator: (1: Yes, 2: No, 3:Unknown)

**Child health: (Most information contained in the inclusion questionnaire to the main trial)**

Chronic conditions, e.g. asthma, heart disease or malformations:____(1:Yes, 2: No, 3: Unknown) If yes, which one:_____________________________________________________

Has your child ever had wheezing or whistling in the chest at any time in the past:___(1: Yes, 2: No) Has your child ever been coughing at any time in the past:___(1:Yes, 2: No, 3: Unknown)

If yes, when was the last time:____/_____/_____

Did your child experience coughing during the last 4 weeks:___(1: Yes, 2: No)

**Current status: (Most information contained in the inclusion questionnaire to the main trial)**

| Maternal medication in the past week: | |  |  |
| --- | --- | --- | --- |
| Antibiotics | ___________________ | Anti-malarials __________________________ | |
| Paracetamol | ___________________ | Other | __________________________ |

Nasal swap performed? YES NO

Signature of mother/caretaker: _______________________________________

BHP worker completing this form: ____________________________________

**The effect of early measles vaccine on pneumococcal colonization**

**Follow-up 6½ months**

**General information:**

Date of follow-up: __/___/____ Study identification number:___________________

Child’s name:____________________________________________ Sex:___ (1: Male, 2: Female) Child’s date of birth:___/_____/_____

Mother/guardian name: ____________________________________

Accepts to participate in follow-up investigation at 6½ months:__ (1:Yes, 2: No)

**Clinical history:**

Child is well at the moment:___ (1: Yes, 2: No)

Has the child been hospitalised or going to consultation since it was enrolled into the study:____

(1: Yes, 2: No, 3: Unknown)

Where 1:__________________ Date 1:___/___/___ Why 1:_________________________

Where 2:__________________ Date 2:___/___/___ Why 2:_________________________

Where 3:__________________ Date 3:___/___/___ Why 3:_________________________

Did your child experience coughing since it was enrolled:___

(1: Yes, 2: No, 3 don’t nkow)

Mother is still breastfeeding:___(1:Yes, 2: No)

If no, when did she stop breastfeeding:____/____/____

**Immunisations**

| **Vaccine** |  |  | **Date** |
| --- | --- | --- | --- |
|  |  |  |  |
| BCG |  |  |  |
|  |  |  |  |
| OPV at birth |  |  |  |
|  |  |  |  |
| Penta1 |  |  |  |
|  |  |  |  |
| OPV1 |  |  |  |
|  |  |  |  |
| Penta2 |  |  |  |
|  |  |  |  |
| OPV2 |  |  |  |
|  |  |  |  |
| Penta3 |  |  |  |
|  |  |  |  |
| OPV3 |  |  |  |
|  |  |  |  |
| Measles Vaccine |  |  |  |
|  |  |  |  |
| **Campaigns** |  |  |  |
|  | Date 1 | Date 2 | |
| Vitamin A: | ____/_____/____ |  | ____/____/____ |
| Polio: | ___/_____/_____ |  | ____/____/____ |
| Measles: | ___/_____/_____ |  | ____/____/____ |
| Other: _______________ | ___/_____/_____ |  | ____/____/____ |
| **Current status:** |  |  |  |

According to the mother, does the child have

Diarrhea (1:Yes, 2: No, 3: Don’t know)

Fever (1:Yes, 2: No, 3: Don’t know)

Cough (1:Yes, 2: No, 3: Don’t know)

Other symptoms (1:Yes, 2: No, 3: Don’t know)

| Child medication in past 3 days: | |  |  |
| --- | --- | --- | --- |
| Antibiotics | ___________________ | Anti-malarials __________________________ | |
| Paracetamol | ___________________ | Other | __________________________ |
| Maternal medication in the past week: | |  |  |
| Antibiotics | ___________________ | Anti-malarials __________________________ | |
| Paracetamol | ___________________ | Other | __________________________ |

**Physical examination**

Does the child appear ill:____ (1: Yes, 2: No)

| Weight :___________kg | Height:__________cm | Arm circumf:____________mm |
| --- | --- | --- |
| Temperature_______C° | Respiration frequency:_______ _/min | |
| Nasal swap performed at 6½ months follow-up?YES | | NO |

Signature of mother/caretaker: _______________________________________

BHP worker completing this form: ____________________________________

**The effect of early measles vaccine on pneumococcal colonization**

**Follow-up 9 months**

**General information:**

Date of follow-up: __/___/____ Study identification number:___________________

Child’s name:____________________________________________ Sex:___ (1: Male, 2: Female) Child’s date of birth:___/_____/_____

Mother/guardian name: ____________________________________

Accepts to participate in follow-up investigation at 9 months: (1:Yes, 2: No)

**Clinical history: (Most information contained in the questionnaire to the main trial)**

Child is well at the moment:___ (1: Yes, 2: No)

Has the child been hospitalised or going to consultation since it was enrolled into the study:____

(1: Yes, 2: No, 3: Unknown)

Where 1:__________________ Date 1:___/___/___ Why 1:_________________________

Where 2:__________________ Date 2:___/___/___ Why 2:_________________________

Where 3:__________________ Date 3:___/___/___ Why 3:_________________________

Has your child had wheezing or whistling in the chest since last visit:___(1: Yes, 2: No)

Has your child been coughing since last visit:___(1:Yes, 2: No, 3: Unknown) If yes, when was the last time:____/_____/_____

| Child medication in past 3 days: | |  |  |
| --- | --- | --- | --- |
| Antibiotics | ___________________ | Anti-malarials __________________________ | |
| Paracetamol | ___________________ | Other | __________________________ |
| Maternal medication in the past week: | |  |  |
| Antibiotics | ___________________ | Anti-malarials __________________________ | |
| Paracetamol | ___________________ | Other | __________________________ |
| Nasal swap performed at 9 months follow-up?YES | | | NO |

Signature of mother/caretaker: _______________________________________

BHP worker completing this form: ____________________________________
