# Supplementary material for: Effect of early measles vaccine on pneumococcal colonization: A randomized trial from Guinea-Bissau
Source: PLoS One. 2017 May 17;12(5):e0177547. doi: 10.1371/journal.pone.0177547 (PMC5435222; doi:10.1371/journal.pone.0177547)
Supplement: S2 Protocol — Estudo dos Efeitos não Específicos da Vacina do Sarampo na presença de S.pneumoniae nas vias aéreas nasais das Crianças Guineenses. (DOCX) [file pone.0177547.s006.docx]

**S2. Protocol in Portuguese**

**Estudo dos Efeitos não Específicos da Vacina do Sarampo na presença de S.pneumoniae nas vias aéreas nasais das Crianças Guineenses.**

**Investigadores:**

Cesário Martins (Médico, PhD)

Nadja Skadkær Hansen (Estudante de Medicina)

Morten Bjerregaard-Andersen (Médico)

Christine S. Benn (Médica, PhD, DMSc)

Peter Aaby (DMSc)

Hilton Whittle (Médico, Professor)

**Local de Realização do Estudo:**

Projecto de Saúde de Bandim, Bissau, Guiné-Bissau (Centros de Saúde de Bandim, Belém e Cuntum).

Serum Staten Institut, Copenhaga, Dinamarca

**Endereço:**

Peter Aaby, Director

Projecto Saúde Bandim

Apartado 861

1004 Bissau Codex, Guiné-Bissau

Email: p.aaby@bandim.org

**Índice**

1. Introdução, *(pag 3)*.
2. Justificativo, (*pag 4*).
3. **Objectivos**, **(***pag 5*).
4. **Metodologia do Estudo**, **(***pag 5*).

4.1 Local do Estudo, ***(****pag 5)*.

4.2 População do Estudo, ***(****pag 5)*.

4.3 Desenho do Estudo, ***(****pag 6)*.

4.4 Métodos utilizados, ***(****pag 7)*.

4.5 Critérios de Inclusão, ***(****pag 7)*.

4.6 Critérios de Exclusão, ***(****pag 8)*.

4.7 Resultados a medir, ***(****pag 8)*.

4.8 Análise de Resultados, ***(****pag 8)*.

4.9 Tamanho da Amostra, ***(****pag 8)*.

1. **Duração do Estudo**, ***(****pag 9)*.
2. **Divulgação dos Resultados**, ***(****pag 9)*.
3. **Considerações Éticas**, ***(****pag 10)*.
4. **Lista de Referências**, ***(****pag 11)*.

**Anexos**

1. **Informação para Consentimento Informado**, ***(****pag 12)*.
2. **Consentimento Informado**, ***(****pag 12)*.
3. **Questionários**, ***(****pag 14)*.
4. **Responsável do Estudo e da Instituições Envolvidas**, ***(****pag 18)*.
5. **Orçamento**, ***(****pag 18)*.

# Introdução

Diversos estudos observacionais em vários países [^1^](#_ENREF_1)^,^[^2^](#_ENREF_2) e um Ensaio randomizado na Guiné-Bissau[^3^](#_ENREF_3) têm demonstrado os efeitos benéficos da vacina do Sarampo (MV) na redução da mortalidade infantil, um efeito que é independente do seu efeito directo que é a prevenção do Sarampo. No ensaio randomizado o rácio da taxa de mortalidade em crianças que foram vacinadas aos 4,5 e aos 9 meses comparativamente com a das crianças que foram apenas vacinadas aos 9 meses foi de 0.70 (0.52-0.94), e independente do efeito protector para o Sarampo[^3^](#_ENREF_3). A análise de um subgrupo de crianças demonstrou que aquelas que tinham ainda anticorpos maternos para o Sarampo no momento da primeira vacinacao tiveram um acentuado incremento na taxa de sobrevivência (MRR 0.17; 0.05-0.58) comparativamente com crianças onde já não existiam anticorpos maternos a quando da vacinação. (Aaby et al, submetido).

A análise dos internamentos hospitalares demonstrou uma redução significativa de internamentos por pneumonia/afecções respiratórias em crianças vacinadas precocemente para o Sarampo. O rácio do risco de admissão foi de 0.37 (0.37; 0.16-0.89) comparativamente com o grupo controlo (Aaby et al, submetido). Esta descoberta foi recentemente suportada num estudo Dinamarquês em que a vacina sarampo-papeira-rubélula foi associada com uma redução de 21% (95% CI 17-24%) no risco de infecções respiratórias (Sørup et al, submetido). Deve ser notado que estes resultados foram obtidos num contexto de ausência de epidemias de Sarampo, ou seja, são independentes do efeito específico de proteção para o Sarampo.

Estudos preliminares na Gambia sugerem que a vacina para o Sarampo pode reduzir a presenca de *S. pneumoniae* nas vias nasofaríngicas. Estas observações estão em concordância com estudos de laboratório que evidenciam que um patogéneo pode induzir uma imunidade hieróloga e dessa forma reduzir a susceptibilidade a infecções subquentes com outro patógeno não relacionado[^4^](#_ENREF_4)^,^[^5^](#_ENREF_5).

Globalmente as infecções por *S. pneumoniae* são uma das principais causas de morbilidade e mortalidade pediátrica; estima-se que *S. pneumoniae* é anualmente responsável por mais de 800,000 mortes e 15 milhões infecções graves, incluindo pneumonia, em crianças com menos de 5 anos, sendo que 90% dos casos ocorre em países em vias de desenvolvimento[^6^](#_ENREF_6). Embora seja assintomática, a colonização nasofaríngica é considerada o primeiro passo no processo da infecção[^7^](#_ENREF_7). Crianças em contextos de baixo rendimento económico apresentam muito cedo e de forma abundante a presença de *S. pneumoniae*[^8^](#_ENREF_8). Existem vacinas específicas para estas bactérias que previnem infecções invasivas, mas estas não foram ainda implementadas em muitos países pobres, incluindo a Guiné-Bissau.

# Justificativo

No projecto de Saúde Bandim, um grande ensaio randomizado para avaliar o impacto dos anticorpos maternos nos efeitos não específicos das vacinas está em progresso. Normalmente as criancas são vacinadas para o Sarampo aos 9 meses de idade. Neste ensaio, as crianças são randomizadas 2:1 para duas doses de vacina, sendo uma tomada aos 4,5 meses e a segunda aos 9 meses. Uma colheita de sangue é feita a todas as crianças para posterior pesquisa da presença de anticorpos maternos. A mortalidade e admissões hospitalares serão avaliadas entre os dois grupos nos períodos: 4 aos 8 meses e dos 9 aos 36 meses. Os resultados vão ser analisados de acordo com a presença de anticorpos maternos contra o Sarampo aos 4,5 meses para testar a hipóteses que a vacinação precoce é particularmente benéfica quando é dada na presença de anticorpos maternos.

Propomos que o mecanismo pelo qual a vacinação precoce para o Sarampo tem um efeito benéfico não específico nas infecções respiratórias reduzindo a mortalidade e o risco de admissão hospitalar, pois anula ou reduz a presença de *S.pneumoniae* nas vias respiratórias

# Objectivo

Dentro do contexto do ensaio principal, desenhamos este estudo para testar a hipótese que a vacinação precoce com a vacina do sarampo reduz o risco da colonização por *S.pneumonia*e Queremos verificar se a vacinação aos 4,5 meses com a vacina do Sarampo esta associado com o risco de presença de *S. pneumoniae* nas vias respiratórias aos 6.5 e 9 meses de idade.

# Metodologia do Estudo

- 1. Local do Estudo

Projecto de Saúde de Bandim, Bissau, Guiné-Bissau (Centros de Saúde de Bandim, Belém e Cuntum). O Projecto de Saúde Bandim (BHP) implementou um sistema de vigilância demográfica em 6 bairros da cidade, cobrindo mais de 102,000 indivíduos em 6 subúrbios. A todos os residentes é atribuído um número de identificação único e informação demográfica e de saúde é recolhida. Todas as casas na area coberta pelo BHP são visitadas mensalmente por um assistente que regista novas gravidezes e nascimentos. Quando um recém nascido é registado, a criança é seguida trimestralmente até aos três anos de idade. Informações sobre o estado de saúde e vacinação são registadas a cada visita.

- 1. População do Estudo

Este estudo está inserido num estudo mais vasto sobre a vacinação precoce para o Sarampo, um ensaio randomizado que foi iniciado em Agosto 2011 e que continuará a recrutar crianças até uma amostra global de 6600 crianças seja alcançada, o que esperamos aconteça em finais de 2014. O objectivo primário deste grande estudo é avaliar o efeito da vacinação precoce para o Sarampo na criança até estas atingirem os 36 meses de idade. Todas as crianças recebem a dose recomendada de vacina para o Sarampo aos 9 meses de idade. Um subgrupo de crianças que participam neste grande estudo serão convidados a participar neste estudo específico sobre *S. pneumoniae*

- 1. Desenho do Estudo

Num subgrupo de crianças vamos testar o efeito da vacinação precoce para o Sarampo na presença e proliferação de *S. pneumoniae* nas vias aéreas nasofaringicas. Assim, em 420 crianças que receberam a vacinação precoce e 210 controlos vai ser realizado um esfregaço com zaragatoa na região nasofaríngica, realizado imediatamente antes da randomização, 2 meses depois, e novamente aos 9 meses de idade. O esfregaço é realizado imediatamente antes da vacinação para o Sarampo. A presença de *S. Pneumonia* vai ser posteriormente determinada nas amostras (Figura 1).


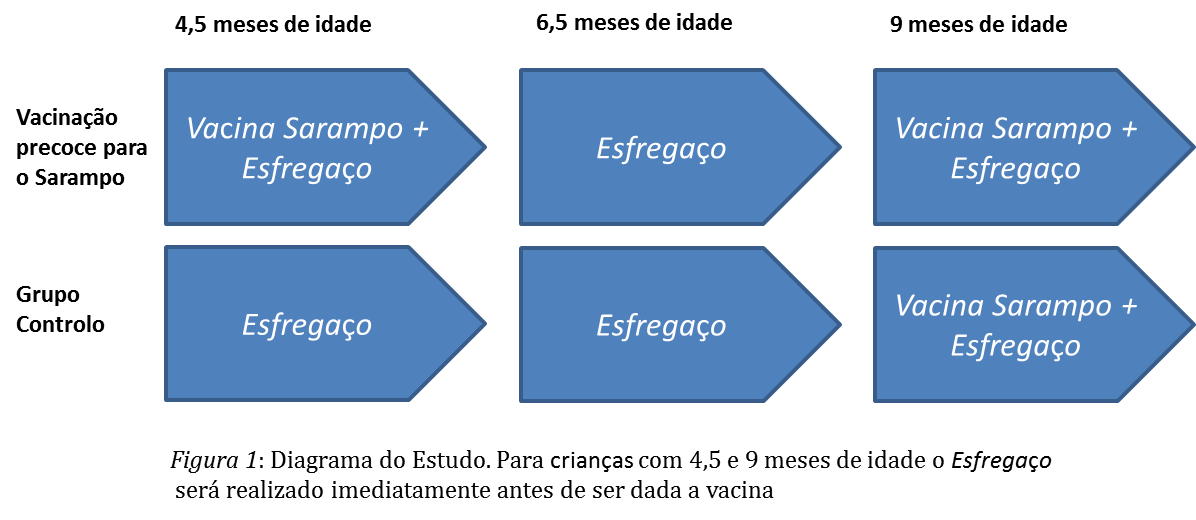


O estudo vai ser realizado pela estudante de medicina Nadja Skadkaer, Universidade de Copenhaga. Peter Aaby (DMSc), Christine S. Benn (MD, DMSc) and Cesário Martins (MD,PhD) que são Investigadores no Ensaio de Vacinação precoce para o Sarampo e serão supervisores neste subestudo. Hilton Whittle, que desenhou o presente estudo sobre S.Pneumoniae será o supervisor sénior do mesmo. Morten Bjerregaard-Andersen (MD) é Investigador Post-Doc no CVIVA estando colocado no Projecto de Saúde Bandim na Guiné Bissau. Morten Bjerregaard-Andersen já trabalhou anteriormente em estudos bacteriológicos e vai apoiar a estudante Nadja Skadkaer na execução do estudo. As análises PCR serão realizadas no Departamento de Microbiologia e Controlo de Infecções do Statens Serum Institut, em colaboração com Jørgen Skov e outros.

- 1. **Métodos utilizados**
- Esfregaço Naso-faríngico

Uma pequena zaragatoa flexível é inserida numa das narinas da criança até aproximadamente metade da distância entre a ponta do nariz e o lobo da orelha[^9^](#_ENREF_9)^,^[^10^](#_ENREF_10). A zaragatoa e esfregaço recolhido serão então inseridos num recipiente numerado contendo em meio de cultura apropriado: *“1 ml skim milk-tryptone-glucose-glycerin (STGG)”* e transportado em gelo para o laboratório num período máximo de três horas. Todos os recipientes serão conservados a -40°C até serem transportados para o *Statens Serum Institut*, Dinamarca, onde serão processados

- *PCR*

A técnica PCR será realizada para medir a quantidade total de *S. pneumoniae* usando um método quantitativo em tempo real adaptado de Corless[^11^](#_ENREF_11)

- 1. **Critérios de Inclusão**

Os únicos critérios de Inclusão são:

- a criança participou no Estudo “Dose adicional da vacina de sarampo aos 4 meses de idade para reduzir a mortalidade infantil e explorar o papel dos anticorpos maternos de Sarampo nos efeitos benéficos não específicos (NSE) na vacina de Sarampo”
- o consentimento informado foi obtido.

- 1. **Critérios de Exclusão**
- A criança não participou no estudo “Dose adicional da vacina de sarampo aos 4 meses de idade para reduzir a mortalidade infantil e explorar o papel dos anticorpos maternos de Sarampo nos efeitos benéficos não específicos (NSE) na vacina de Sarampo”
- o consentimento informado não foi concedido..
  1. **Resultados a medir**

Prevalência de S. Pneumoniae nas vias aéreas nasais das crianças avaliadas.

- 1. **Análise de Resultados**

O Software utilizado para análise de dados será o Stata. As característica de base dos diferentes grupos serão comparadas usando *Testes Chi Quadrado* para dados de contingência e *Testes T* para variáveis contínuas. Análises de regressão multivariada do *Modelo de Poisson*, com variância robusta serão aplicadas no cálculo das taxas de prevalência usadas para avaliar o efeito da vacinação precoce para o Sarampo sendo os resultados estratificados por potenciais modificadores desse efeito: sexo e presença de anticorpos maternos. As diferenças serão consideradas significativas a partir de P<0.05.

- 1. **Tamanho da Amostra**

A prevalência de portadores de *S. pneumoniae* na Guiné-Bissau é desconhecida, mas baseando-nos em dados de áreas singulares[^12^](#_ENREF_12)^,^[^13^](#_ENREF_13) assumimos que a prevalência no grupo de controlo será de 85% com 420 crianças no grupo de intervenção e 210 crianças no grupo de controlo, será possível demonstrar uma redução de 85% para 75% associada com a vacinação precoce para o Sarampo com um poder de 80% (p<0.05). Decidimos manter o rácio 2:1 para explorar futuramente o papel potencial dos anticorpos maternos.

1. **Duração do Estudo**

As amostras serão realizadas pelo estudante de medicina Nadja Skadkær Hansen e um médico local, cada um ajudado por um assistente local. O trabalho será supervisionado pelos responsáveis do estudo da vacina contra sarampo: O Director Nacional do Projecto Saúde Bandim, Cesário Martins, e também Morten Bjerregaard-Andersen, Christine Stabell Benn e Peter Aaby. Os PCR serão realizados no Departamento de Microbiologia e Controlo de Infecções do Statens Serum Institut em colaboração com Steen Hoffmann e Jørgen Skov Jensen.

**Cronograma de estudo**

Este estudo está planeado decorrer entre Agosto 2013 e Julho 2014:

| Data |  |
| --- | --- |
| 09/2013 | Preparação e formação da Equipa. Leitura da literatura de fundo. Introdução ao Projecto. |
| 10/2013-05/2014 | Supervisão da colecta de dados e entrada de dados. Apresentação do projecto para outros pesquisadores e alunos do PSB. |
| 06/2014-07/2014: | Análise de amostras, análise de dados e interpretação. Preparação do relatório científico. Divulgação dos resultados |

1. **Divulgação dos Resultados**

Será importante saber se os efeitos benéficos da vacinação para o Sarampo na mortalidade global e nas infecções respiratórias é mediada por um efeito na prevenção/redução da presença de S. *pneumoniae* nas vias respiratórias. Adicionalmente o estudo vai fornecer informação sobre a prevalência de S. *pneumoniae* nas criancas da Guiné Bissau. Um estudo recente associou a presença de *S. pneumoniae* com défice no crescimento [^8^](#_ENREF_8); (este resultado pode também ser testado com este estudo).

Os resultados serão publicados em revistas internacionais. As ligações estreitas dos parceiros com os formuladores de políticas nacionais de saúde vão assegurar que o conhecimento se traduz em políticas de saúde sem atrasos desnecessários.

Uma cópia do Relatórios Final do estudo, traduzida em português, será entregue na Biblioteca do INASA, e às Direcções Regionais de Saúde e ao Ministério da Saúde Guineense.

1. **Considerações Éticas**

O protocolo do estudo da vacinação precoce para o Sarampo foi aprovado pelos Comités de ética da Guiné-Bissau e Dinamarca. Este protocolo será submetido a aprovação pelas mesmas entidades. As crianças inscritas no presente estudo já se inscreveram no ensaio da vacinação precoce para o Sarampo. Ressaltamos que a participação neste estudo é voluntária. As crianças são submetidas a um esfregaço nasofaríngico indolor, são pesadas, medidas e respondem a um questionário (ver Anexos). O teste não tem risco envolvido para as crianças. A pessoa que executa testes é um médico ou estudante de medicina treinados para o efeito.

O estudo e seus objectivos vão ser explicados em crioulo às mães das crianças consideradas elegíveis. Quando a mãe não estiver disponível, o pai ou tutor serão procurados. O procedimento é explicado da seguinte forma: "O seu filho foi incluído num estudo de vacina contra sarampo. Nós gostaríamos de testar se vacina contra sarampo tem um efeito protector para afecções respiratórias provocadas por *S. pneumoniae*. O teste envolve fazer um esfregaço com zaragatoa no interior do nariz da criança. Gostaríamos também de medir e pesar o seu filho e fazer algumas perguntas sobre a sua saúde. Ao participar neste projecto irá nos ajudar a aprender sobre os efeitos da vacina contra sarampo e pode ajudar as crianças no futuro. O procedimento do teste é muito seguro. A criança vai sentir apenas um desconforto momentâneo.". Será fornecido mediante um Consentimento Informado escrito em Português (documento em anexo). Se o pai ou o tutor concordar, o documento de consentimento será lido e assinado.

1. **Lista de Referências**
2. Aaby, P. *et al.* Non-specific beneficial effect of measles immunisation: analysis of mortality studies from developing countries. *BMJ* **311**, 481-485 (1995).
3. Aaby, P. *et al.* The optimal age of measles immunisation in low-income countries: a secondary analysis of the assumptions underlying the current policy. *BMJ Open* **2**, doi:10.1136/bmjopen-2011-000761 (2012).
4. Aaby, P. *et al.* Non-specific effects of standard measles vaccine at 4.5 and 9 months of age on childhood mortality: randomised controlled trial. *BMJ* **341**, c6495 (2010).
5. Welsh, R. M. & Selin, L. K. No one is naive: the significance of heterologous T-cell immunity. *Nat Rev Immunol* **2**, 417-426, doi:10.1038/nri820 (2002).
6. Kleinnijenhuis, J. *et al.* Bacille Calmette-Guerin induces NOD2-dependent nonspecific protection from reinfection via epigenetic reprogramming of monocytes. *Proceedings of the National Academy of Sciences of the United States of America* **109**, 17537-17542, doi:10.1073/pnas.1202870109 (2012).
7. O'Brien, K. L. *et al.* Burden of disease caused by Streptococcus pneumoniae in children younger than 5 years: global estimates. *Lancet* **374**, 893-902, doi:10.1016/S0140-6736(09)61204-6 (2009).
8. Bogaert, D., De Groot, R. & Hermans, P. W. Streptococcus pneumoniae colonisation: the key to pneumococcal disease. *Lancet Infect Dis* **4**, 144-154, doi:10.1016/S1473-3099(04)00938-7 (2004).
9. Coles, C. L. *et al.* Pneumococcal carriage at age 2 months is associated with growth deficits at age 6 months among infants in South India. *J Nutr* **142**, 1088-1094, doi:10.3945/jn.111.156844 (2012).
10. O'Brien, K. L., Nohynek, H. & World Health Organization Pneumococcal Vaccine Trials Carriage Working, G. Report from a WHO Working Group: standard method for detecting upper respiratory carriage of Streptococcus pneumoniae. *Pediatr Infect Dis J* **22**, e1-11, doi:10.1097/01.inf.0000049347.42983.77 (2003).
11. Kaltoft, M. S., Skov Sorensen, U. B., Slotved, H. C. & Konradsen, H. B. An easy method for detection of nasopharyngeal carriage of multiple Streptococcus pneumoniae serotypes. *Journal of microbiological methods* **75**, 540-544, doi:10.1016/j.mimet.2008.08.010 (2008).
12. Corless, C. E. *et al.* Simultaneous detection of Neisseria meningitidis, Haemophilus influenzae, and Streptococcus pneumoniae in suspected cases of meningitis and septicemia using real-time PCR. *J Clin Microbiol* **39**, 1553-1558, doi:10.1128/JCM.39.4.1553-1558.2001 (2001).
13. Roca, A. *et al.* Effects of community-wide vaccination with PCV-7 on pneumococcal nasopharyngeal carriage in the Gambia: a cluster-randomized trial. *PLoS medicine* **8**, e1001107, doi:10.1371/journal.pmed.1001107 (2011).
14. Coles, C. L. *et al.* Newborn vitamin A supplementation does not affect nasopharyngeal carriage of Streptococcus pneumoniae in Bangladeshi infants at age 3 months. *J Nutr* **141**, 1907-1911, doi:10.3945/jn.111.141622 (2011).

**Anexos**

1. **Informação para Consentimento Informado**

O Projecto de Saúde Bandim está a investigar se uma vacinação precoce para o sarampo influencia o risco de colonização das vias aéreas nasais com a bactéria *S.pneumoniae*. Anteriormente a sua criança participou num estudo de vacinação precoce para o Sarampo.

Queremos verificar se essa vacina a protegeu da presença da bactéria *S. pneumoniae* nas vias respiratórias. Nem sempre a presença destas bactérias determina doença, mas é importante verificar se a vacina do sarampo, contribuiu para reduzir esse risco.

Para verificar isto, vamos recolher uma amostra do conteúdo do nariz da criança inserindo uma zaragatoa que vai ser esfregada ligeiramente na parede interior do nariz. Vamos também pesar a criança e realizar algumas perguntas sobre o seu estado de saúde geral.

O procedimento do teste é muito simples e seguro. A criança apenas pode sentir algum desconforto momentâneo.

Todas as informações pessoais são estritamente confidenciais. Os resultados do estudo serão relatados internacionalmente mas sem nunca mencionar a identidade dos participantes. Os dados serão mantidos por tempo indeterminado e podem ser utilizados em outras pesquisas pelo Projecto de Saúde de Bandim.

A participação no estudo é totalmente voluntária e a sua decisão de ser ou não incluído no estudo não afecta o seu relacionamento com o Projecto de Saúde de Bandim.

Se você ainda tiver dúvidas ou quiser obter mais informações sobre este estudo, entre em contacto com o Professor Cesário Martins, no Projecto de Saúde de Bandim ou no Apartado 861, 1004 Bissau Codex, Guiné-Bissau.

1. **Consentimento Informado**

Se não tem dúvidas e aceita participar neste estudo, pedimos-lhe que assine o seguinte consentimento:

- Li, ou foi-me lida em voz alta este documento e compreendi todas as informações dadas.
- Tenho consciência todas as informações recolhidas durante o estudo são confidenciais.
- Tomei conhecimento que o objectivo deste teste é avaliar a presença de um microorganismo específico (*S.pneumoniae*) na criança.
- A participação está baseada no meu total consentimento. Eu posso deixar de participar no estudo a qualquer momento. A recusa de participação no estudo não compromete a minha relação com o Projecto de Saúde de Bandim.
- Foi-me dada a oportunidade de fazer qualquer pergunta a respeito do presente estudo e da minha participação. As perguntas foram respondidas satisfatoriamente.
- A minha participação é voluntária e com base na minha decisão soberana.

Nome da criança_________________________________________________________________________________________

Nome da mãe ou tutor __________________________________________________________________________________

Assinatura ou impressão digital ________________________________¬¬ Data |__|__|-|__|__|-|2|0| _ |__|

Eu _________________________________________________________(nome), Assistente do Projecto de Saúde de Bandim, declaro que expliquei o estudo e suas implicações para o participante no estudo. O participante compreendeu o estudo e suas implicações e deu o seu consentimento em participar.

Assinatura ________________________________________________________ Data |__|__|-|__|__|-|2|0| _ |__|

1. **Questionários**

**3.1 Questionário de Inclusão**

**Informação Geral:**

Bairro:________ Zona:_________ Casa:__________ CNO:____________________ Data de inclusão: __/__/__ Numero de

Numero de identificação – estudo de sarampo: MV________________

Identificação no estudo (“numest”): ____

Nome da Criança: _______________________________________________ Sexo: __ (1:Macho, 2:Fêmea)

Data de Nascimento da criança: __/__/__

Nome da Mãe/Responsável: __________________________________________ Etnia: _____________

Parentesco com a Criança: __ (1:Mãe, 2:Pai, 3:Tia, 4:Avó, 5:Outro)

Numero de telemóvel Mãe/Responsável: ____________________________ 🡪 Relação com a criança: ________________ Aceitou participar no estudo: __ (1:Sim, 2:Não)

Número de irmãos menores de cinco anos____

**Dados socioeconómicos:**

Mãe sabe ler e escrever: __ (1:Sim, 2:Não, 3: Desconhecido)

Estado Civil: __ (1:Solteira, 2:Casada, 3:Divorciada, 4:Viúva,5:Desconhecido)

Educação da Mãe: __ (1:Primária, 2:Secundária, 3: Liceu, 4:Universidade, 5: não estudou, 6: Desconhecido)

Casa tem água canalizada: __ (1:Sim, 2:Não, 3:Desconhecido)

Casa tem televisão: __ (1:Sim, 2:Não, 3:Desconhecido)

Casa tem frigorífico: __ (1:Sim, 2:Não, 3:Desconhecido)

**Informações sobre a exposição ao tabaco**

A Mãe fuma cigarros? ____ (1: Sim, 2: Nao) Se sim, fuma quando está perto da criança? ____ (1: Sim, 2: Não)

Qualquer pessoa em contato diário com a criança fuma cigarros quando está com a criança?___ (1: Sim, 2: Não)

A casa onde a criança vive tem uma lareira / pequena cozinha que produz fumaça? ____ (1: Sim, 2: Não)

Se sim, é dentro ou fora de casa? ____ (1: Interior, 2: Fora de Casa) Que material é queimado? ___________________________

**Saúde da Crianca:**

A Crianca tem doenças crónicas (ex: Asma ou malformações cardíacas: __ (1:Sim, 2:Não, 3:Desconhecido)

Se sim, indique qual: ____________________________________________________

A sua criança já alguma vez teve sibilos no peito: __ (1:Sim, 2:Não)

A sua criança já alguma vez teve tosse: __ (1:Sim, 2:Não, 3:Desconhecido)

Se sim, quando foi a última vez: __/__/__

A sua criança esteve com tosse nas últimas 4 semanas: __ (1:Sim, 2:Não)

**Situação actual:**

Indique a Medicação Maternal na última semana:

Antibióticos ⃝ ____________________ Antimaláricos ⃝ ____________________

Paracetamol ⃝ ____________________ Outros ⃝ ____________________

Foi realizado o esfregaco nasal ? SIM ⃝ NÃO ⃝ ⃝ Hora: _____:_____

Se não, indique porquê:____________________________________________________________

*Nome do funcionário PSB que efectuou o esfregaco nasal:____________________________________________*

Antes de efectuar o Esfregaço o nariz da criança estava bloqueado com secreções? ___ (1:Sim,2:Nao)

*Nome do funcionário PSB que efectuou o questionário:___________________________________________*

Assinatura da Mãe/Responsável pela criança:

_________________________________________________________ __________________________________________________

Assinatura do funcionário BHP que efectuou o questionário:

_____________________________________________________________________ ______________________________________

**3.2 Questionário de Follow-up (6,5 meses)**

**Informação Geral:**

Data de follow-up: __/__/__ Numero de identificação no estudo (“numest”): ____

Nome da Criança: _______________________________________________ Sexo: __ (1:Macho, 2:Fêmea)

Data de Nascimento da criança: __/__/__

Nome da Mãe/Responsável: __________________________________________

Aceitou responder a este questionário: __ (1:Sim, 2:Não)

**História Clínica**

Criança está bem de saúde: __ (1:Sim, 2:Não)

A criança foi internada ou foi ao médico desde o início do estudo: __ (1:Sim, 2:Não, 3:Desconhecido)

Se sim, indique:

Onde1: ______________________________ Data1 :__/__/__ Motivo1 :_______________________

Onde2: ______________________________ Data2 :__/__/__ Motivo2 :_______________________

Onde3: ______________________________ Data3 :__/__/__ Motivo3 :_______________________

A criança teve tosse em algum momento desde o início do estudo: __ (1:Sim, 2:Não, 3:Desconhecido)

A mãe ainda amamenta a criança: __ (1:Sim, 2:Não)

Se não, quando parou de amamentar: __/__/__

**Imunizações**

| Vacina | Data |
| --- | --- |
| BCG | __/__/__ |
| OPV (Nascimento) | __/__/__ |
| Penta 1 | __/__/__ |
| OPV 1 | __/__/__ |
| Penta 2 | __/__/__ |
| OPV 2 | __/__/__ |
| Penta 3 | __/__/__ |
| OPV 3 | __/__/__ |
| Sarampo | __/__/__ |

**Campanhas**

| Campanha | Data 1 | Data 2 |
| --- | --- | --- |
| Vitamina A | ⃝ __/__/__ | ⃝ __/__/__ |
| Polio | ⃝ __/__/__ | ⃝ __/__/__ |
| Sarampo | ⃝ __/__/__ | ⃝ __/__/__ |
| Outra | ⃝ __/__/__ | ⃝ __/__/__ |

Situação actual:

De acordo com a Mãe ou responsável pela criança , esta está com:

Diarreia: __ (1:Sim, 2:Não, 3:Desconhecido)

Febre: __ (1:Sim, 2:Não, 3:Desconhecido)

Tosse: __ (1:Sim, 2:Não, 3:Desconhecido)

Outros sintomas de doença: __ (1:Sim, 2:Não, 3:Desconhecido)

Indique quais:________________________________________________

Indique a medicação da criança nos últimos 3 dias:

Antibióticos ⃝ ____________________ Antimaláricos ⃝ ____________________

Paracetamol ⃝ ____________________ Outros ⃝ ____________________

⃝ Não tomou medicação

Indique a Medicação Maternal na última semana:

Antibióticos ⃝ ____________________ Antimaláricos ⃝ ____________________

Paracetamol ⃝ ____________________ Outros ⃝ ____________________

⃝ Não tomou medicação

**Observacão do Estado Geral da crianca**

A criança parece estar doente: __ (1:Sim, 2:Não)

Peso: ____ (kg); Altura: ____ (cm); Circunferência do Braço: ____ (mm);

Temperatura: ____(C⁰)

Foi realizado o esfregaço nasal aos 6,5 meses? SIM ⃝ NÃO ⃝

Assinatura da Mãe/Responsável pela criança:

_________________________________________________________ __________________________________________________

Assinatura do funcionário BHP que efectuou o questionário:

_____________________________________________________________________ ______________________________________

**3.3 Questionário de Follow-up (9meses)**

**Informação Geral:**

Data de follow-up: __/__/__ Numero de identificação no estudo (“numest”): ____

Nome da Criança: _______________________________________________ Sexo: __ (1:Macho, 2:Fêmea)

Data de Nascimento da criança: __/__/__

Nome da Mãe/Responsável: __________________________________________

Aceitou responder a este questionário: __ (1:Sim, 2:Não)

**História Clínica:**

Criança está bem de saúde: __ (1:Sim, 2:Não)

A criança foi internada ou foi ao médico desde o início do estudo: __ (1:Sim, 2:Não, 3:Desconhecido)

Se sim, indique:

Onde1: ______________________________ Data1 :__/__/__ Motivo1 :_______________________

Onde2: ______________________________ Data2 :__/__/__ Motivo2 :_______________________

Onde3: ______________________________ Data3 :__/__/__ Motivo3 :_______________________

A sua criança teve sibilos no peito desde a última visita: __ (1:Sim, 2:Não)

A sua criança esteve com tosse desde a última visita: __ (1:Sim, 2:Não, 3:Desconhecido)

Se sim, quando foi a última vez: __/__/__

Indique a medicação da criança nos últimos 3 dias:

Antibióticos ⃝ ____________________ Antimaláricos ⃝ ____________________

Paracetamol ⃝ ____________________ Outros ⃝ ____________________

⃝ Não tomou medicação

Indique a Medicação Maternal na última semana:

Antibióticos ⃝ ____________________ Antimaláricos ⃝ ____________________

Paracetamol ⃝ ____________________ Outros ⃝ ____________________

⃝ Não tomou medicação

Foi realizado o esfregaço nasal aos 9 meses? SIM ⃝ NÃO ⃝

Assinatura da Mãe/Responsável pela criança:

_________________________________________________________ __________________________________________________

Assinatura do funcionário BHP que efectuou o questionário:

_____________________________________________________________________ ______________________________________

1. **Responsável do Estudo e Instituições Envolvidas**

O Professor Peter Aaby é o Fundador Director do Projecto de Saúde de Bandim, com mais de 30 anos de experiência nos estudos epidemiológicos e ensaios clínicos na Guiné-Bissau. O Professor Aaby licenciou-se em Antropologia . O Professor Aaby é autor de mais de 480 artigos científicos publicados em revistas internacionais, tendo sido orientador de mais de 33 Doutoramentos (PhD) e de inúmeros estágios de investigação de estudantes de Medicina. Desde 2012 Peter Aaby é professor adjunto da Southern Danish University. Actualmente é ainda co-organizador do CVIVA, um centro de investigação de excelência de vacinas e vitaminas da Fundação Nacional Dinamarquesa para a Investigação.

O estudo é desenvolvido no âmbito do Projecto de Saúde de Bandim (www.bandim.org) na Guiné-Bissau. O Projecto Saúde Bandim é hoje, provavelmente, o maior, e cientificamente mais produtivo, centro de vigilância demográfica e Epidemiológica na África Lusófona, cobrindo 1/3 da capital Bissau e cerca de 250 localidades em todas as regiões da Guiné Bissau. O Projecto de Saúde Bandim é membro fundador do INDEPTH, uma rede internacional de instituições de investigação vigilância demográfica e de saúde contínua em paíes em vias de desenvolvimento. È ainda membro da rede dinamarquesa Internacional de investigação em saúde.O PSB é parceiro da “[*Copenhagen School of Global Health*](http://globalhealth.ku.dk/)” e trabalha em colaboração estreita com a Universidade de Aarhus, *University of Southern Denmark* e várias outras instituições na Dinamarca e outros países.

O Statens Serum Institut é uma entidade pública Dinamarquesa com mais de 100 anos de existência. Faz parte do Ministério da Saúde Dinamarquês e é um centro de referencia mundial de Investigação e Diagnóstico. (www.ssi.dk).

1. **Orçamento**

| Estudante de Medicina | Financiada por Universidade de Copenhaga |
| --- | --- |
| PCR | Realizado no SSI |
| Médico (tarde) | 1.500.000 |
| 2 Assistentes de Zona | 5.700.000 |
| Material para recolha de Amostras | 2.254.000 |
| Computador para lançamento de dados | 500.000 |
| **Total (CFA)** | **9.954.000** |
